# Supplementary figures and images for: Engineering of a compact, high-fidelity EbCas12a variant that can be packaged with its crRNA into an all-in-one AAV vector delivery system
Source: PLoS Biol. 2024 May 30;22(5):e3002619. doi: 10.1371/journal.pbio.3002619 (PMC11139299; doi:10.1371/journal.pbio.3002619)

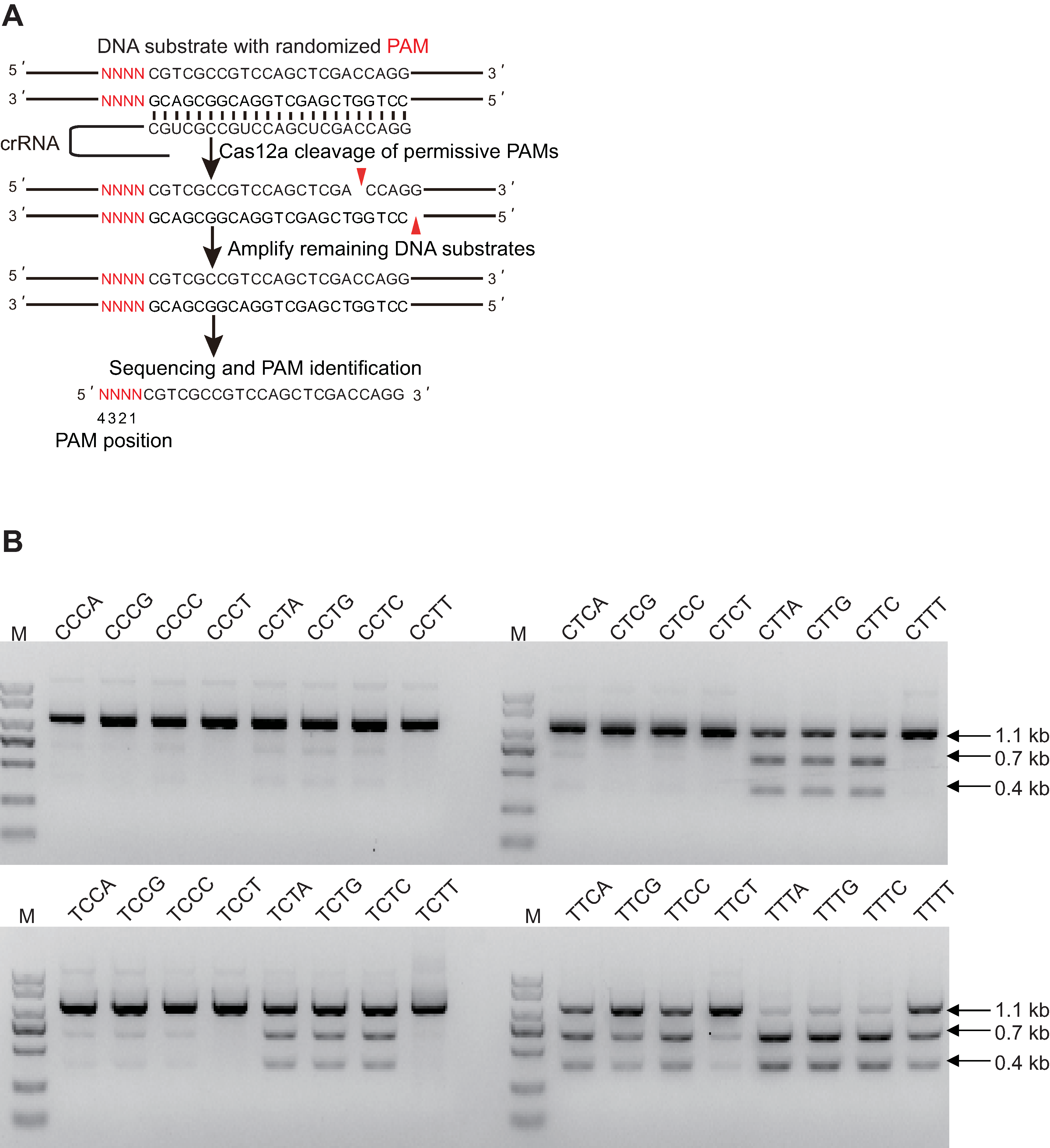

Supplement: S1 Fig — (A) Schematic of in vitro cleavage assay used to identify PAM sequence. (B) The preferences of EbCas12a toward different PAMs in vitro. The EbCas12a-crRNA complex (100 nM) was incubated at 37°C for 8 min with 300 ng DNA substrates with the different PAMs (CCCN, CCTN, CTCN, CTTN, TCCN, TCTN, TTCN, TTTN), respectively. The data underlying this figure can be found in S1 Data. (TIF) [file pbio.3002619.s001.tif]

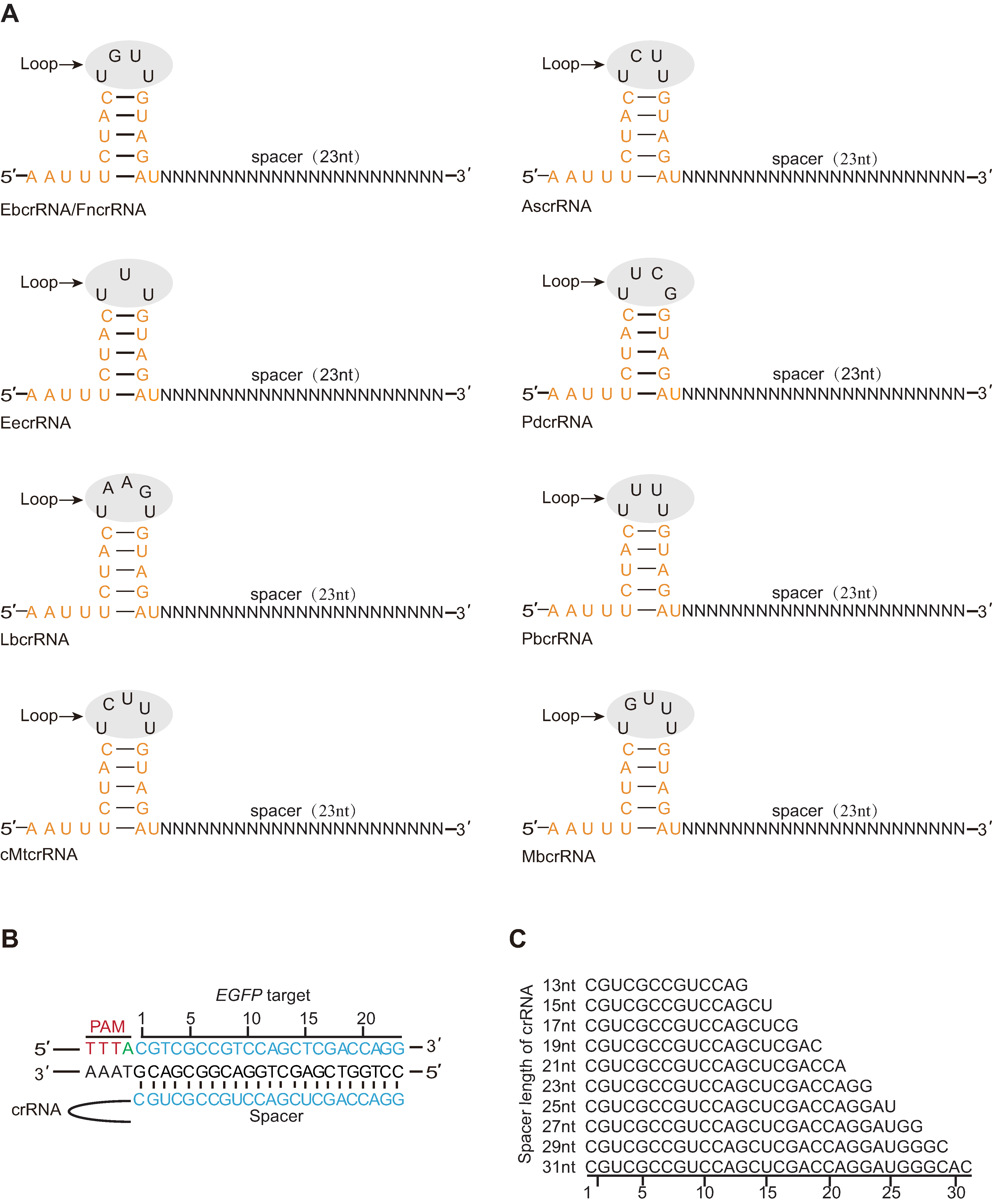

Supplement: S2 Fig — (A) Schematic representation crRNA direct repeat structures. The difference among these 8 Cas12a family members is shown in shadow. (B) Schematic showing the sequence of EGFP-targeting crRNA. (C) Schematic showing variable length complementarity regions for the target site of EGFP in human cells. (TIF) [file pbio.3002619.s002.tif]

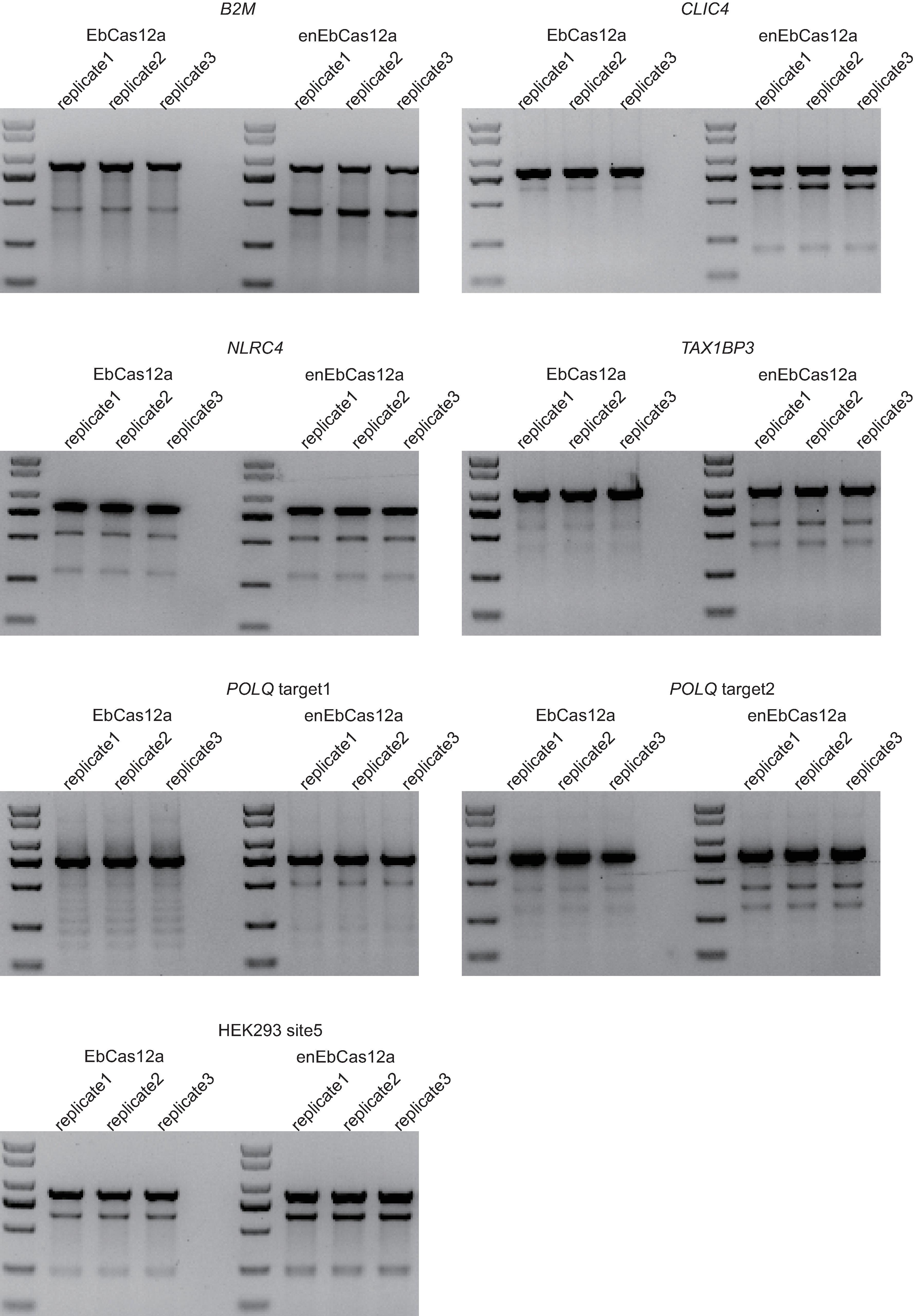

Supplement: S3 Fig — Gel image of Fig 3C. Assessment of gene-editing efficiencies with enEbCas12a. Activities assessed by T7E1 assay. Replicates represented transfected cell cultures times (n = 3). The data underlying this figure can be found in S1 Data. (TIF) [file pbio.3002619.s003.tif]

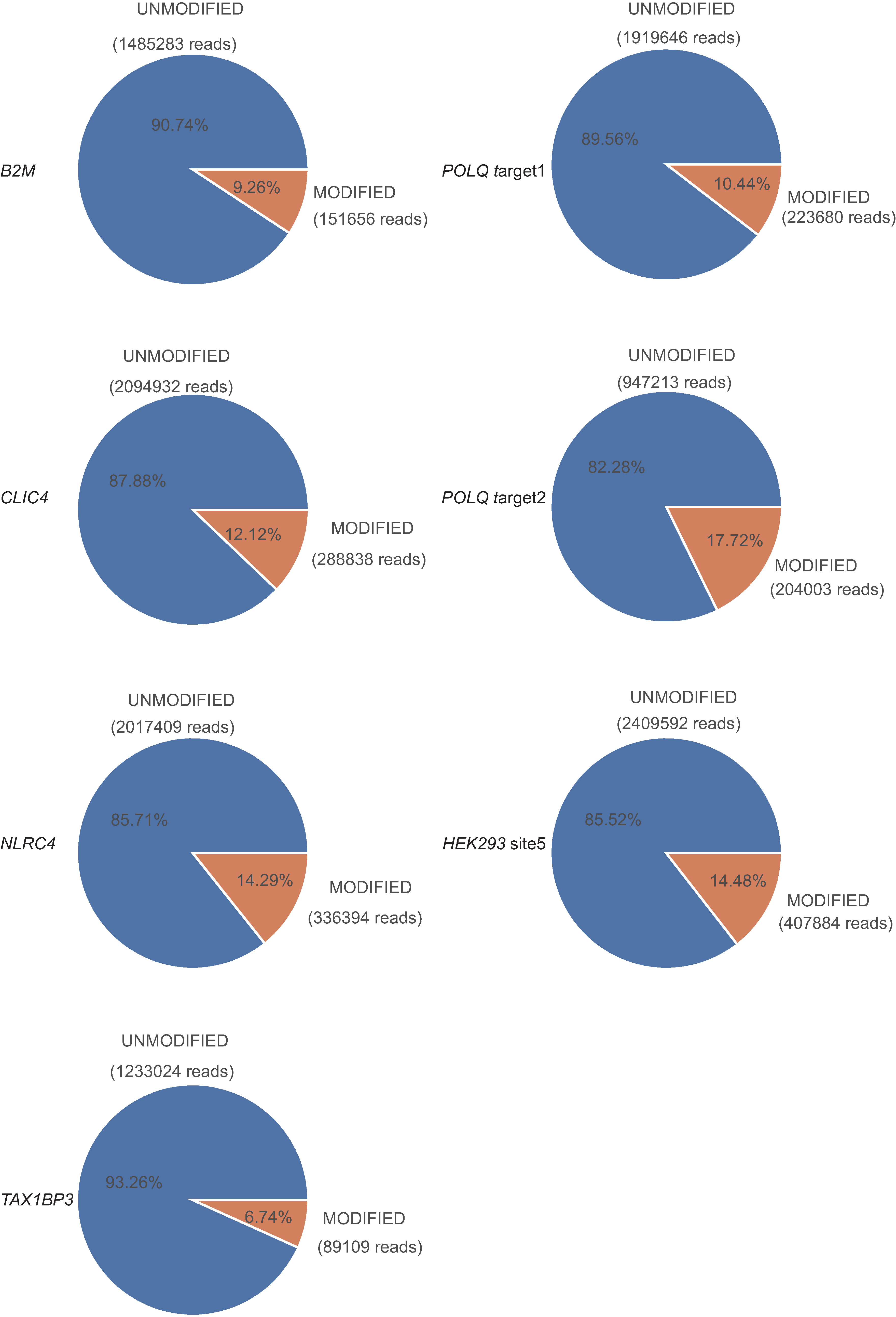

Supplement: S4 Fig — Pie chart showing the deep sequencing read counts and indel frequencies of 7 endogenous gene targets. (TIF) [file pbio.3002619.s004.tif]

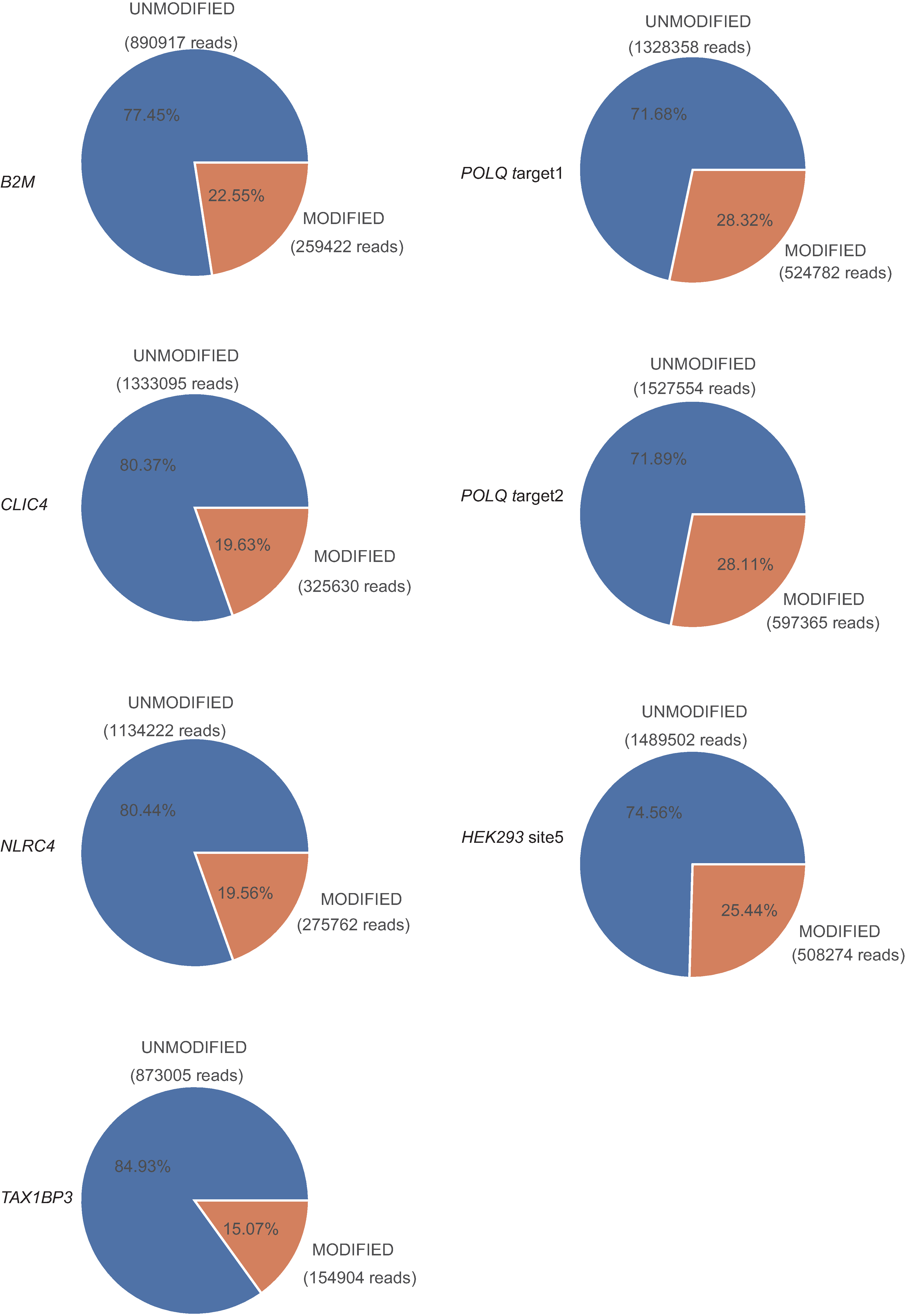

Supplement: S5 Fig — Pie chart showing the deep sequencing read counts and indel frequencies of 7 endogenous gene targets. (TIF) [file pbio.3002619.s005.tif]

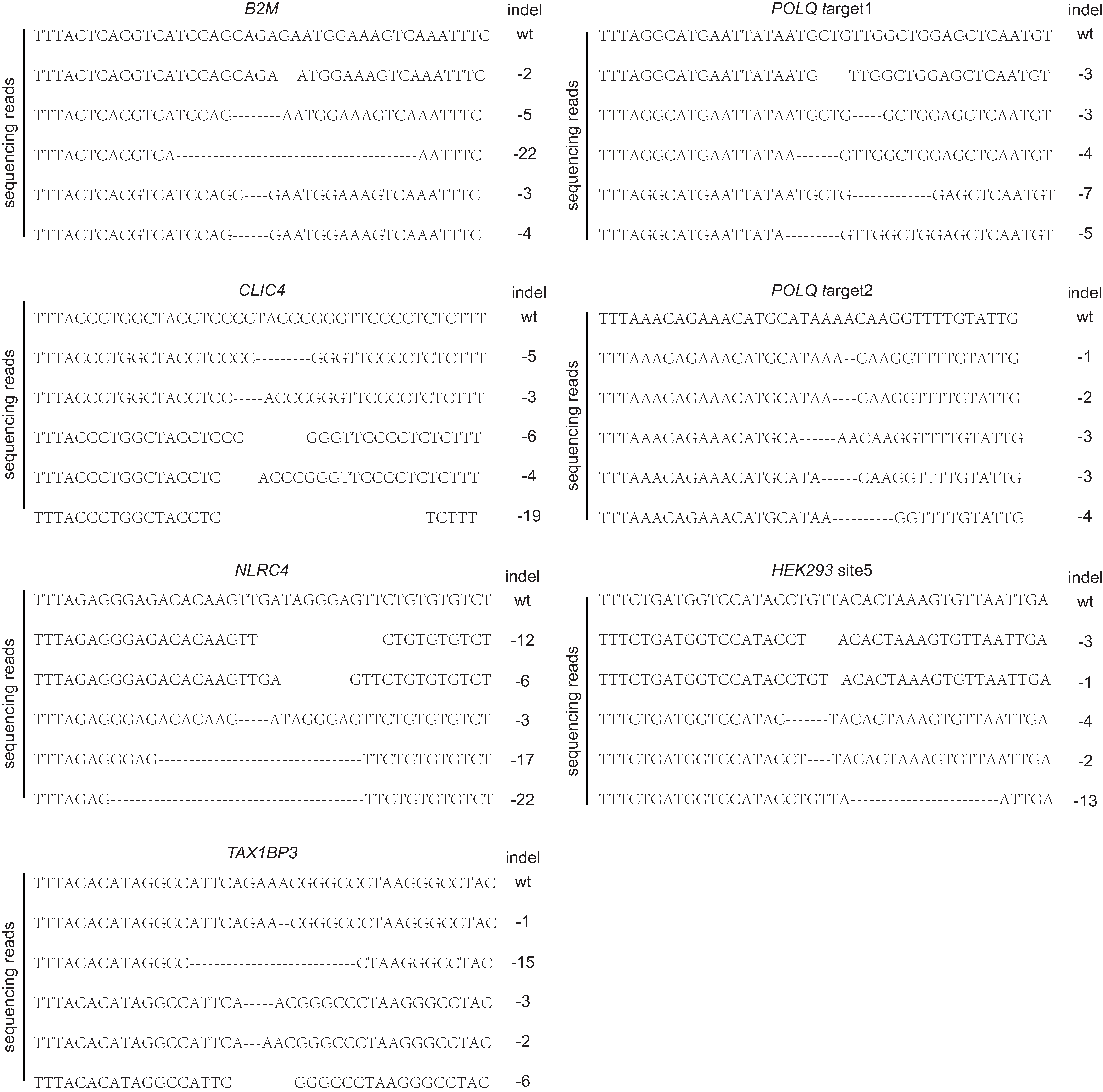

Supplement: S6 Fig — Sequencing reads show representative mutations of enEbCas12a-mediated gene editing with 7 crRNAs. Dashes represent the DNA deletions. The number at the right side of each sequence is the length of indel (−, deletion). (TIF) [file pbio.3002619.s006.tif]

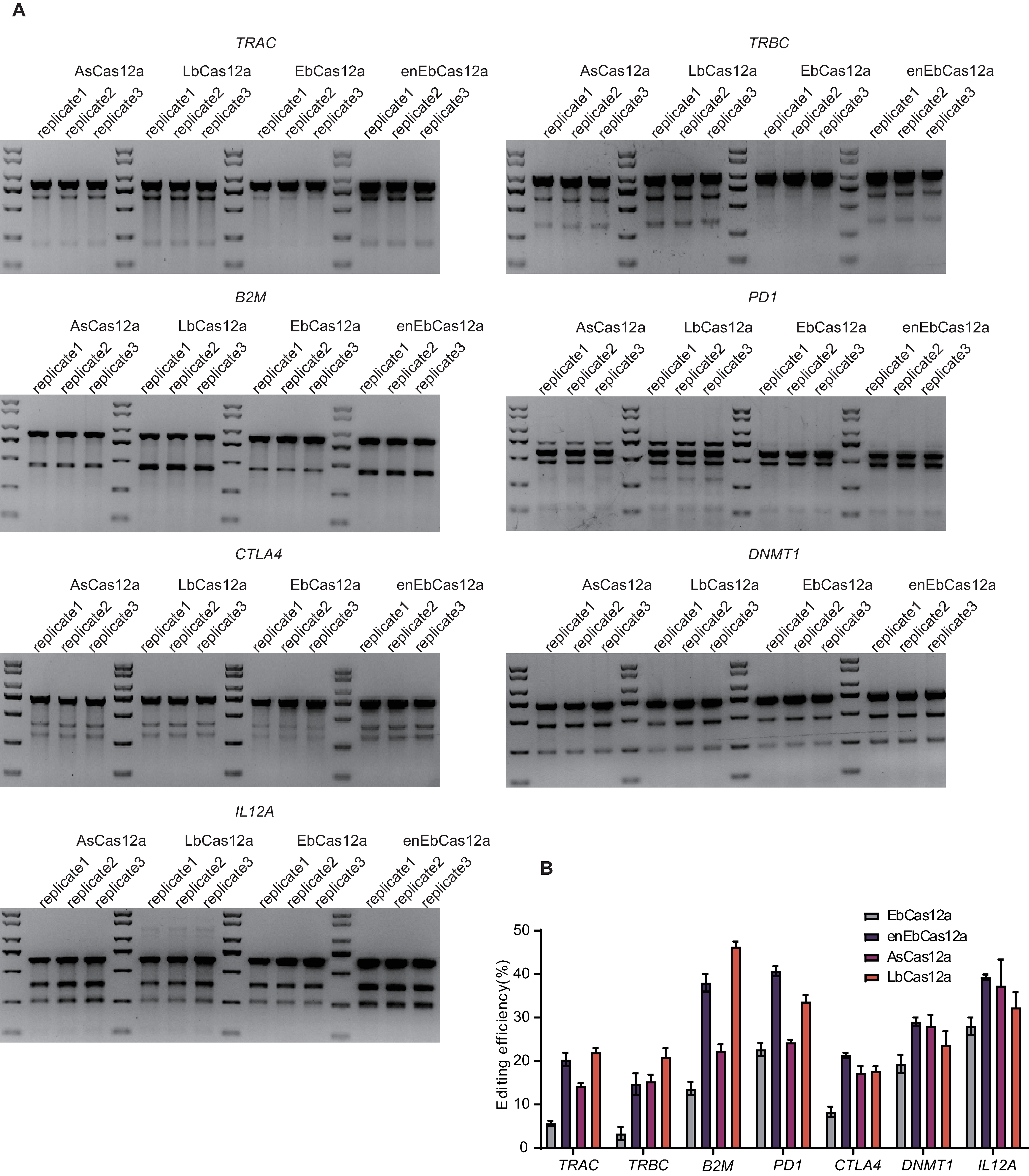

Supplement: S7 Fig — (A) Gel image of T7E1. Replicates represented transfected cell cultures times (n = 3). (B) Indel frequencies analyzed by T7E1 assay. Summaries of the activities of Cas12a at TTTV PAMs from A. The data underlying this figure can be found in S1 Data. (TIF) [file pbio.3002619.s007.tif]

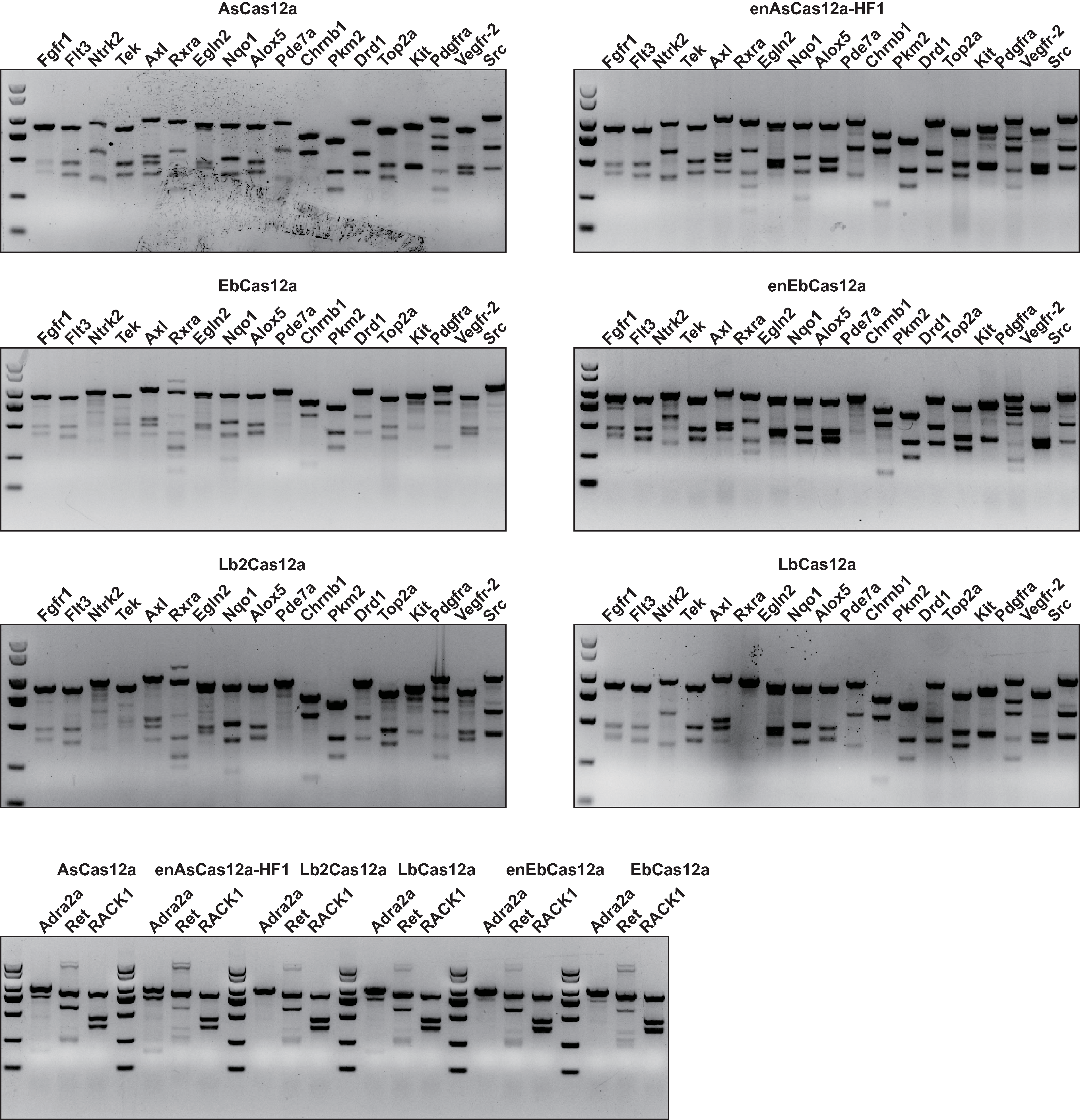

Supplement: S8 Fig — Activity of Cas12a variants on targets with candidate PAM sequences assessed by T7 endonuclease I assay. Average activity of Cas12a variants on targets are shown in Fig 3E. (TIF) [file pbio.3002619.s008.tif]

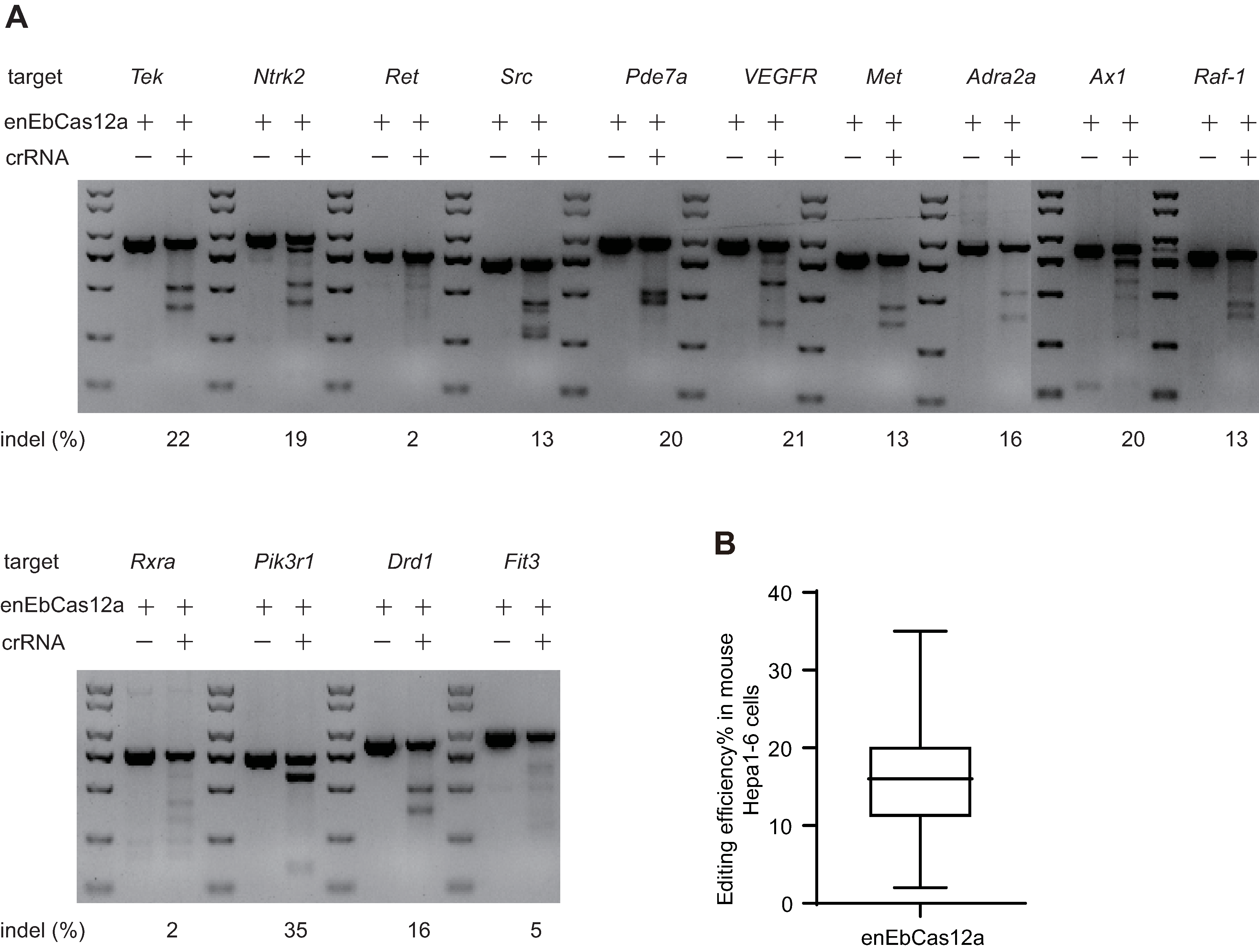

Supplement: S9 Fig — (A) Indel mutations introduced at endogenous gene targets by enEbCas12a at TTTV PAMs. Indel frequencies of 14 gene targets were measured by T7E1 assay. (B) Summaries of the activities of enEbCas12a at TTTV PAMs from A. The data underlying this figure can be found in S1 Data. (TIF) [file pbio.3002619.s009.tif]

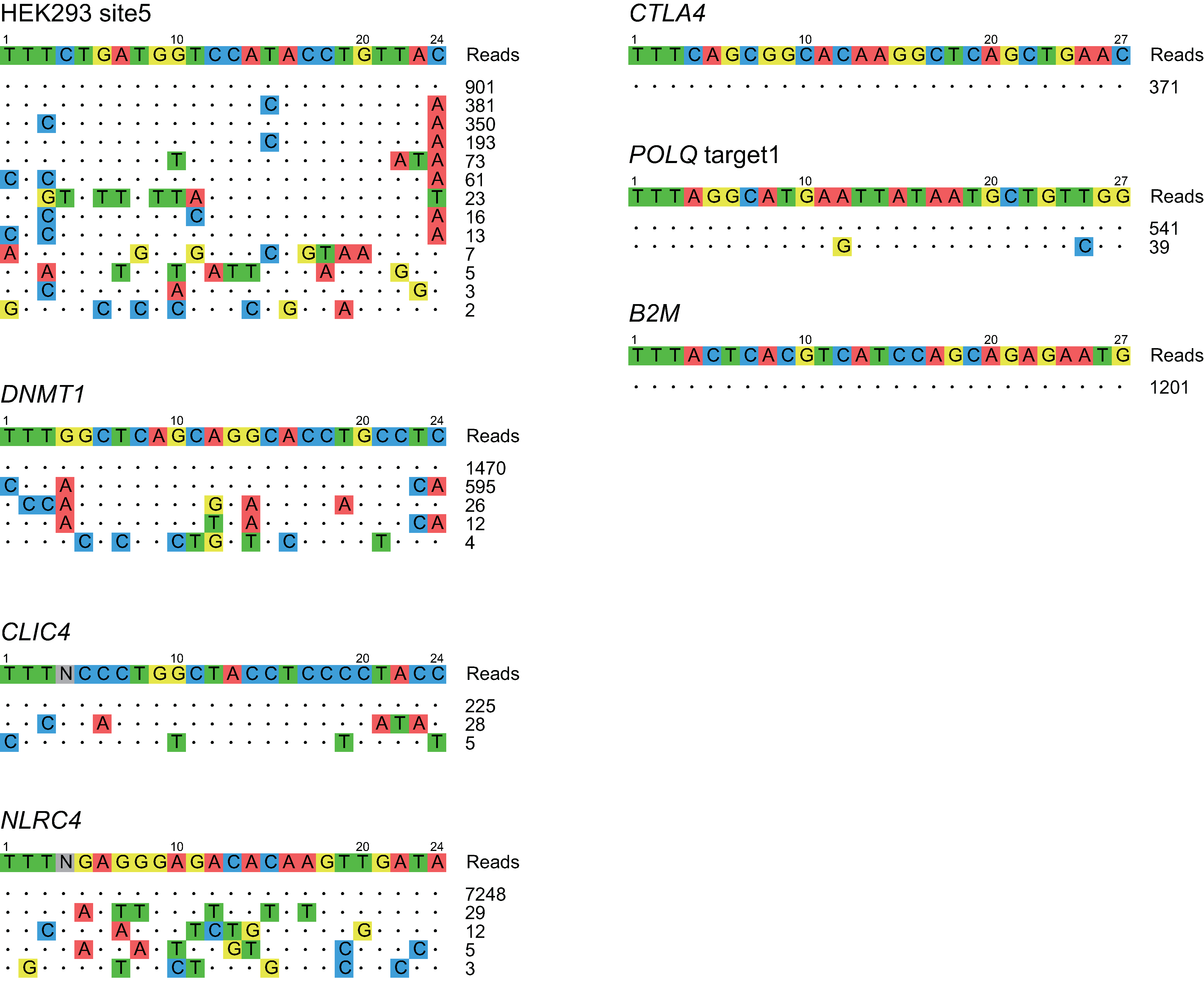

Supplement: S10 Fig — GUIDE-seq analysis of detected off-targets for AsCas12a in Fig 3F. Mismatched positions are highlighted in color, and GUIDE-seq read counts are shown to the right of the on- or off-target sequences. (TIF) [file pbio.3002619.s010.tif]

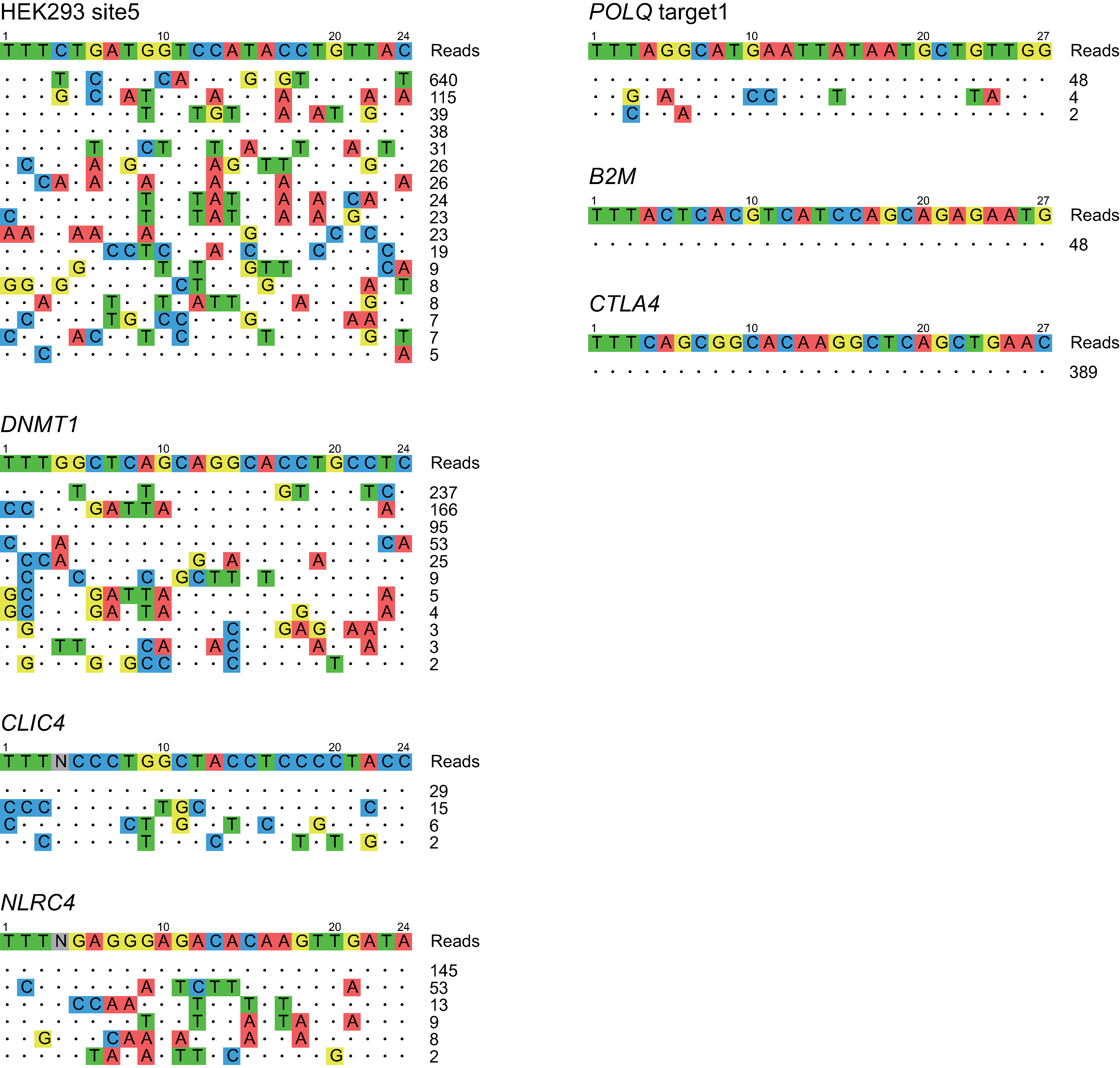

Supplement: S11 Fig — GUIDE-seq analysis of detected off-targets for LbCas12a in Fig 3F. Mismatched positions are highlighted in color, and GUIDE-seq read counts are shown to the right of the on- or off-target sequences. (TIF) [file pbio.3002619.s011.tif]

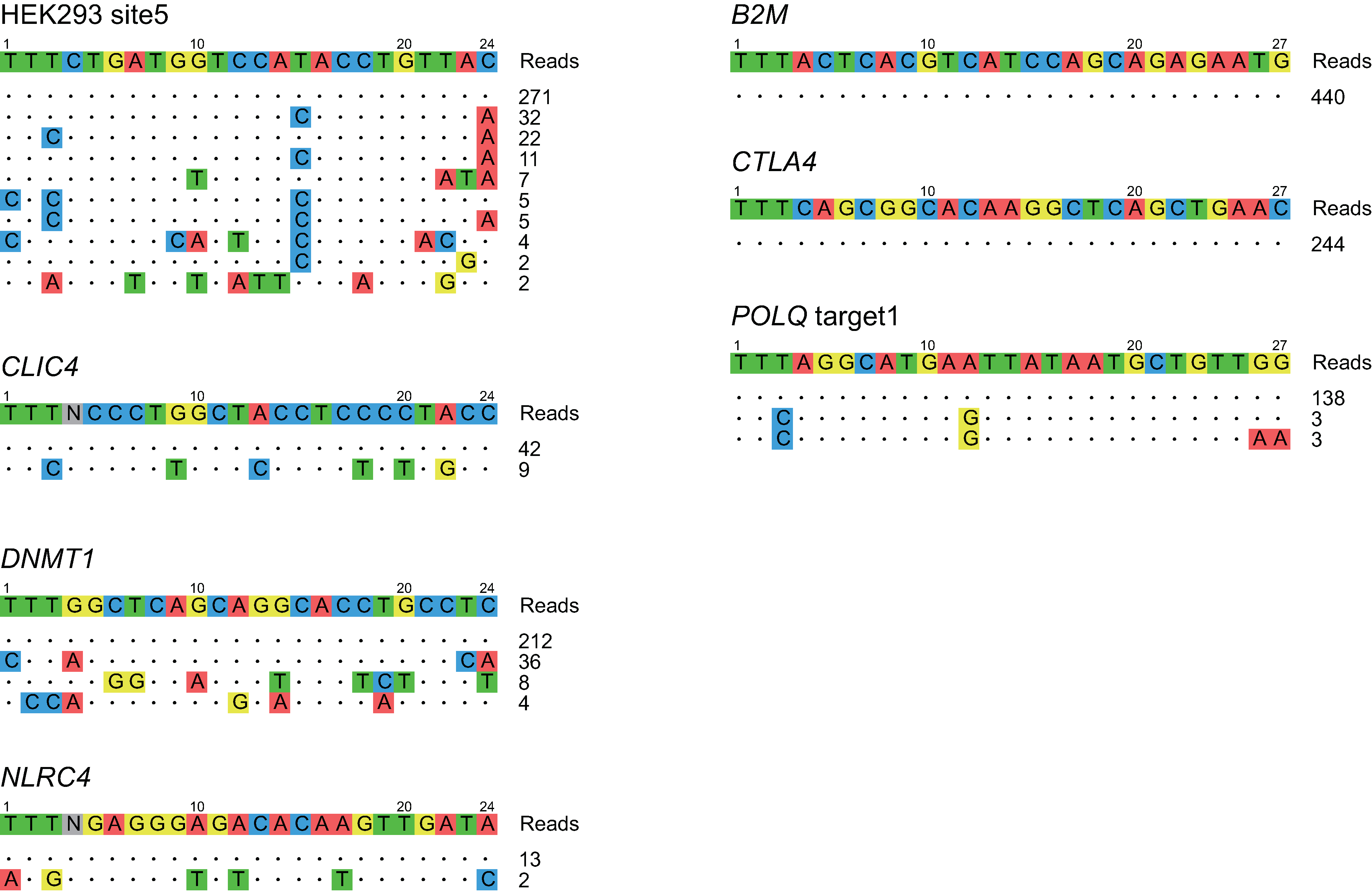

Supplement: S12 Fig — GUIDE-seq analysis of detected off-targets for enEbCas12a in Fig 3F. Mismatched positions are highlighted in color, and GUIDE-seq read counts are shown to the right of the on- or off-target sequences. (TIF) [file pbio.3002619.s012.tif]

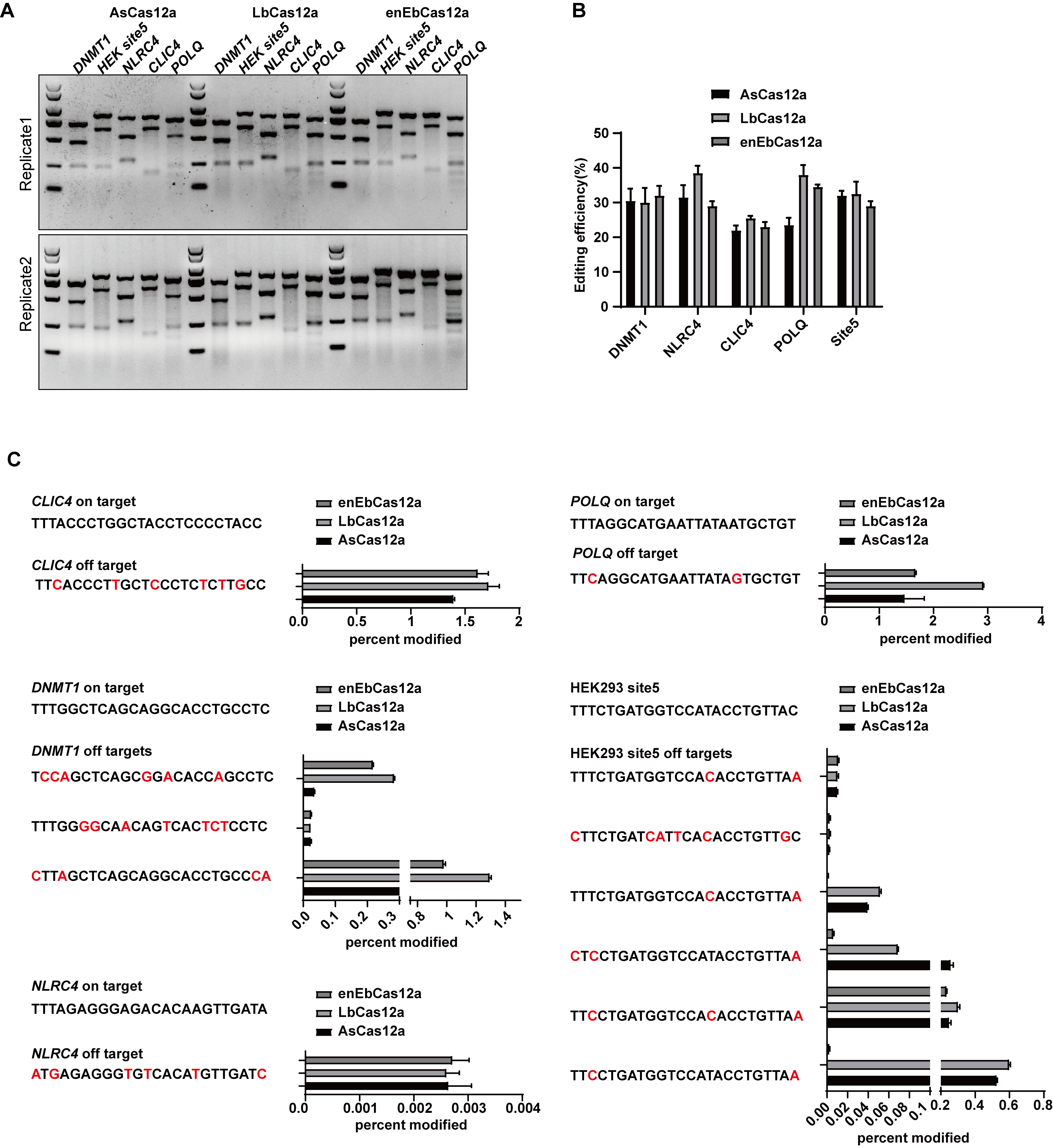

Supplement: S13 Fig — (A) Evaluation of the activity of AsCas12a, LbCas12a, and enEbCas12a. Activity of Cas12a variants on targets assessed by T7 endonuclease I assay. (B) Average activity of Cas12a variants on targets; summary of on-target modifications from A. (C) Percent modification GUIDE-seq detected off-target sites with indel mutations for AsCas12a, LbCas12a, and enEbCas12a. The data underlying this figure can be found in S1 Data. (TIF) [file pbio.3002619.s013.tif]

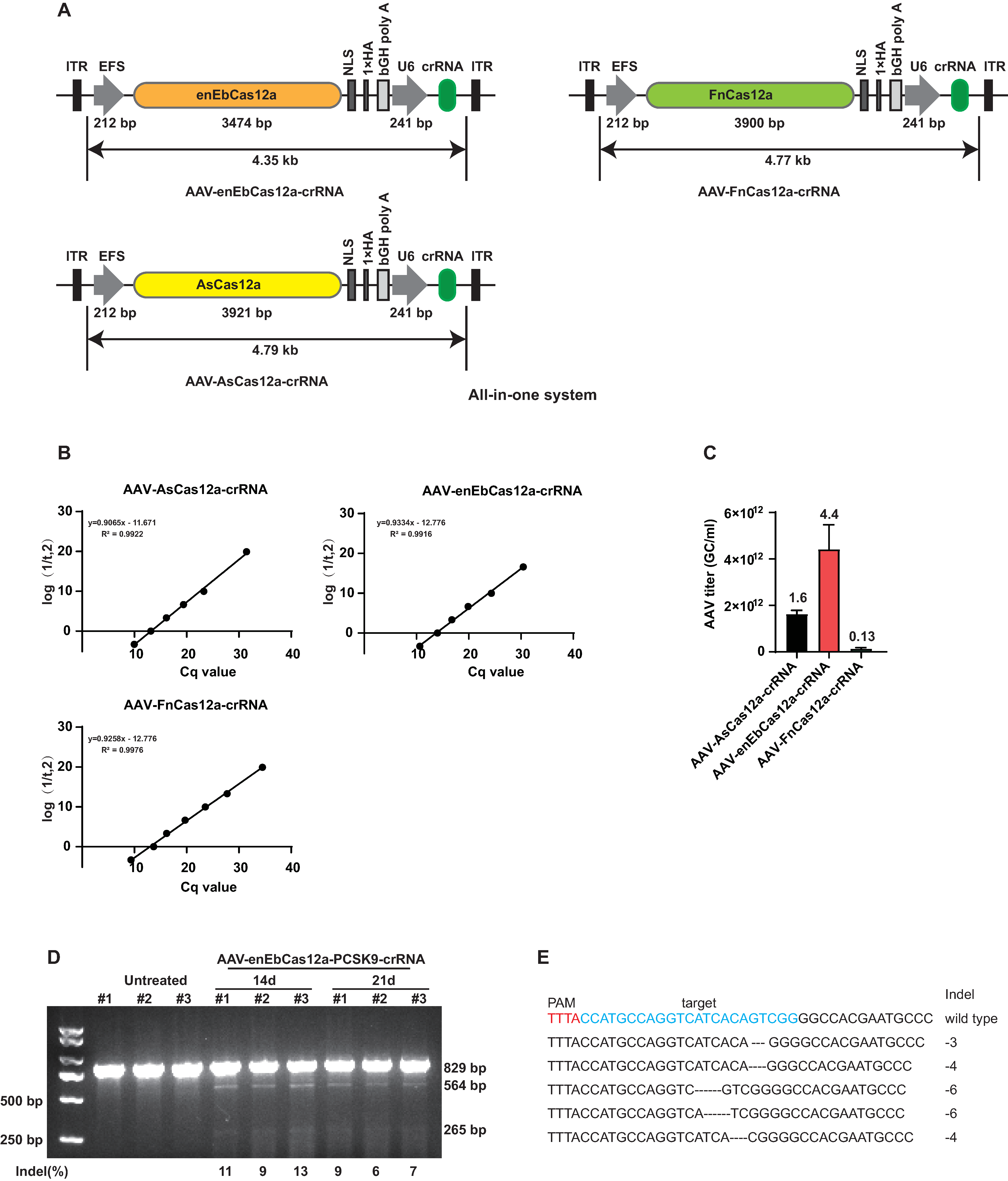

Supplement: S14 Fig — (A) Single vector AAV system. (B) Standard curve showing the data points for the dilution series of the AAV-Cas12a-crRNA plasmid. (C) The titer of AAV-Cas12a-crRNA viral particles. (D) In vivo genome editing using AAV-enEbCas12a-PCSK9-crRNA. Indels analyzed by T7E1 assay (n = 3 animals for time points). (E) Sequencing reads show representative mutations of AAV-enEbCas12a-PCSK9-crRNA-mediated gene editing in liver. Dashes represent the DNA deletions. The number at the right side of each sequence is the length of indel (−, deletion). The data underlying this figure can be found in S1 Data. (TIF) [file pbio.3002619.s014.tif]
