# Supplementary material for: Engineering of a compact, high-fidelity EbCas12a variant that can be packaged with its crRNA into an all-in-one AAV vector delivery system
Source: PLoS Biol. 2024 May 30;22(5):e3002619. doi: 10.1371/journal.pbio.3002619 (PMC11139299; doi:10.1371/journal.pbio.3002619)
Supplement: S1 Table — (DOCX) [file pbio.3002619.s015.docx]

**S1 Table.** List of sequences used in study

| **Target ID** | **PAM** | **Guide sequence (5' to 3')** | **Assay** |
| --- | --- | --- | --- |
| EGFP | TTTA | CGTCGCCGTCCAG (13nt) | EGFP disruption |
| EGFP | TTTA | CGTCGCCGTCCAGCT (15nt) | EGFP disruption |
| EGFP | TTTA | CGTCGCCGTCCAGCTCG (17nt) | EGFP disruption |
| EGFP | TTTA | CGTCGCCGTCCAGCTCGAC (19nt) | EGFP disruption |
| EGFP | TTTA | CGTCGCCGTCCAGCTCGACCA (21nt) | EGFP disruption |
| EGFP | TTTA | CGTCGCCGTCCAGCTCGACCAGG (23nt) | EGFP disruption |
| EGFP | TTTA | CGTCGCCGTCCAGCTCGACCAGGAT (25nt) | EGFP disruption |
| EGFP | TTTA | CGTCGCCGTCCAGCTCGACCAGGATGG (27nt) | EGFP disruption |
| EGFP | TTTA | CGTCGCCGTCCAGCTCGACCAGGATGGGC (29nt) | EGFP disruption |
| EGFP | TTTA | CGTCGCCGTCCAGCTCGACCAGGATGGGCAC(31nt) | EGFP disruption |
| EGFP | NNNN | CGTCGCCGTCCAGCTCGACCAGG | PAM screen |
| PD1 | TTTA | GCACGAAGCTCTCCGATGTGTTG | T7E1 |
| HBB | TTTG | GGGATCTGTCCACTCCTGATGCT | T7E1 |
| IL12A | TTTA | GGATGCCACTAAAAGGGAAAGGG | T7E1 |
| CLIC4 | TTTA | CCCTGGCTACCTCCCCTACC | T7E1/GUIDE-seq |
| NLRC4 | TTTA | GAGGGAGACACAAGTTGATA | T7E1/GUIDE-seq |
| HEK293 site5 | TTTC | TGATGGTCCATACCTGTTACACT | T7E1/GUIDE-seq |
| POLQ target1 | TTTA | GGCATGAATTATAATGCTGTTGG | T7E1/GUIDE-seq |
| POLQ target2 | TTTA | AACAGAAACATGCATAAAACAAG | T7E1 |
| TAX1BP3 | TTTA | CACATAGGCCATTCAGAAACGGG | T7E1 |
| TRBC | TTTG | AGCCATCAGAAGCAGAGATCTCC | T7E1 |
| TRAC | TTTG | TTGCTCCAGGCCACAGCACTGTT | T7E1 |
| CTLA4 | TTTC | AGCGGCACAAGGCTCAGCTGAAC | T7E1/GUIDE-seq |
| Met (Mouse) | TTTA | TCTCGGAGCCACAAACTACATTT | T7E1 |
| Ntrk2 (Mouse) | TTTG | GTGTACTGAGCCTTCTCCAGGCA | T7E1 |
| Ret (Mouse) | TTTG | GGAAGGTGTCGTTGATGAAGGAG | T7E1 |
| Src (Mouse) | TTTC | CGAGGGCTCCAGGCTGCGGCGCC | T7E1 |
| Pde7a (Mouse) | TTTG | ACTGTGGTAATCTTCTTGAATCA | T7E1 |
| VEGFR-2 (Mouse) | TTTC | ATCCCACTACCGAAAGCAATAAA | T7E1 |
| Met (Mouse) | TTTA | TCTCGGAGCCACAAACTACATTT | T7E1 |
| Adra2a (Mouse) | TTTG | CCTGGCTGGCCTGCTCATGCTGT | T7E1 |
| Rxra (Mouse) | TTTC | CTGAGGGATGGGCAGGAACCTTG | T7E1 |
| Pik3r1 (Mouse) | TTTG | GTACGAGACGCATCTACTAAAAT | T7E1 |
| Drd1 (Mouse) | TTTA | GCTGTGTCAGATCTCTTGGTGGC | T7E1 |
| Flt3 (Mouse) | TTTG | GAATAAAAAGGCTAACAGAAAAG | T7E1 |
| Fgfr1 (Human) | TTTC | TCCTCTGAAGAGGAGTCATCATC | T7E1 |
| Flt3 (Human) | TTTA | GGTATGAAAGCCAGCTACAGATG | T7E1 |
| Flt3 (Human) | TTTA | CCCGAAACAAACTGACGAGTTTG | T7E1 |
| Axl (Human) | TTTC | CCTGGCGCAGATAGTCATAAATC | T7E1 |
| Rxra (Human) | TTTG | CCTCCACGTAGGTCTCGGTCTTG | T7E1 |
| Egln1  (Human) | TTTG | GATTATCAACATGACGTACATAA | T7E1 |
| Nqo1 (Human) | TTTG | GGATGAGACACCACTGTATTTTG | T7E1 |
| Alox5 (Human) | TTTC | ACTATCAAACTGGATATCACGGG | T7E1 |
| Pde7a (Human) | TTTC | ACTCCACTGCTTGCTTAATTCCC | T7E1 |
| Chrnb1 (Human) | TTTA | GACGCAGGTACAGCGGAAGTTTG | T7E1 |
| Pkm2 (Human) | TTTG | GGGTCGAGCAGGATGTTGATATG | T7E1 |
| Drd1 (Human) | TTTG | GGTGGGCTAATTCATCCTTGAAC | T7E1 |
| Top2a (Human) | TTTG | GCTGAATCTCCCTCAGTCAGGAT | T7E1 |
| Kit  (Human) | TTTC | ATGTCCATGTACTCATTAGTACT | T7E1 |
| PDGFR-α (Human) | TTTC | CCCTGACTTATACACGGTTTTAA | T7E1 |
| VEGFR-2 (Human) | TTTA | CTATTCCCAGCTACATGATCAGC | T7E1 |
| Src (Human) | TTTC | AGAGGAGCCCATTTACATCGTCA | T7E1 |
| PCSK9 | TTTA | CCATGCCAGGTCATCACAGTCGG | AAV |
| enEbCas12a-NLS | ATGAGCAAGTTCCAGAACCTGTACACCATCAACAAGACCCTGCGCTTCGGCCTGAAGCCCTTCGGCAAGACCCTGGAGAACTTCAACAAGACCAACCTGCTGCAGCTGGACGAGTACAAGGCCAAGCACCGCAAGGAGGTGCAGCGCCTGTTCGACGAGAACTTCAAGCAGCTGATCGAGGAGCGCCTGCGCGGCCTGAGCCTGGACACCCAGGCCCTGGAGGAGGCCTTCGACATCAACAAGCGCGACGCCGCCCTGATCAGCCTGAAGAAGCAGGTGACCGGCATCTGCTACGACAGCGAGATGAAGAAGACCTACCTGCAGGCCGACAAGCACTTCCAGAAGCTGCTGGCCGCCGGCCCCAACCAGGCCATGGTGTGCACCTACGACAAGTTCAGCACCTACTTCGTGAACTTCTTCcgcATCCGCACCCACATCTTCAAGGGCGACACCAGCGGCAGCATCGCCTACCGCCTGATCGACGAGAACCTGACCATCTTCAAGAAGAACGTGGACAAGATCGCCAAGCTGCCCGTGGGCCTGAAGGACGAGGTGGAGGAGCTGGCCGACATCGAGAGCCTGCAGAGCTACAACAGCTACCTGACCCAGAGCGGCATCACCGAGTACAACGAGCTGCTGGGCGGCATCGCCTACGAGGACGGCACCAAGCTGCAGGGCATCAACGAGAAGATCAACCTGTACGGCCAGAAGAACAAGCTGAAGCTGCCCCGCCTGGAGAGCCTGTACAAGATGATCCTGAGCGACCGCGAGACCCAGAGCTTCGTGCTGAGCATCATCGAGAACGACGCCGAGCTGATCGGCCAGATCAGCACCCTGCTGGAGGACGTGCTGCTGAGCAAGACCCTGGCCCTGAGCGACGTGGACGGCGTGTTCATCAAGTACACCCAGCTGGGCAACCTGCCCGGCGTGCCCTACACCGTGATCAACAGCAAGATCAACGAGGCCTTCGACGCCACCTACACCGGCAAGAAGGAGGGCGAGAAGTACAAGGTGACCAAGAAGAAGACCATCGAGAAGGACGTGTACAGCCTGAGCAAGATCGAGAAGCTGTTCCAGGACAGCGACATCAACGTGAGCGAGGCCCTGAAGAACAAGTACGGCGTGCTGATCGCCAGCTACGAGGAGGCCAAGGGCCTGTTCAACAGCATCGACTGGACCGAGATCAAGAACATCAAGCAGAGCGGCCACACCATCATCATCAAGGACGTGCTGGACGCCCTGAAGAACATCCAGTTCTTCTACAACCTGTTCGACGTGGTGGAGGAGAACCTGAACCCCAGCATCGAGTTCTACAACGAGCTGAGCCTGAACAAGAACCAGCTGGGCAACGAGTTCAACAGCACCTACAACAAGGCCCGCAACTACCTGACCAAGAAGGAGTACAGCGAGGAGAAGTTCAAGCTGAACTTCGACAGCCCCACCCTGGCCGACGGCTGGGACGTGAACAAGGAGACCGCCAACCTGACCATCCTGCTGCGCCGCTTCAACAGCGAGCGCAACAACTACGACTACTTCCTGGGCGTGTGGAAGAAGGCCGTGCCCAGCCGCGAGAAGAACCTGATCATCAACGCCGACGGCGAGTTCGAGAAGATGGACTACAAGCTGTACCCCGACCCCAGCAAGATGCTGCCCAAGCAGTTCGTGAGCGCCCAGAGCTGGTTCGACAAGTACCCCGCCAGCCCCGAGTTCATGGGCAAGTACGAGGCCGGCCTGCACAAGAAGGGCAACAACTTCGACATCGAGTTCCTGCACGAGCTGATCAACCGCTACAAGCACGGCCTGAAGCACCACGAGAACAAGTACGAGGAGACCTTCGACTTCGAGCTGAAGGAGACCGAGGAGTACAGCGAGTACAGCGAGTTCATCCAGGACGTGAGCAAGAGCAACTACAAGGTGAAGTTCAACCACGTGGCCGGCGTGGAGGAGCTGGTGGAGGAGGGCAAGCTGTACCTGTTCCAGATCTGGAGCAAGGACTTCAGCACCTTCAGCAAGGGCACCAAGAACCTGAACACCATCTACTTCGAGAGCCTGTTCAGCGAGGAGAACCTGGAGAAGCGCATCTTCAAGCTGAGCGGCGGCGCCGAGCTGTTCTACCGCCCCAAGAGCCTGACCTACACCAAGGAGCTGATGGAGAAGGGCCACCACTACAACGAGCTGAAGGACAGCTTCAACTACCCCATCATCAAGGACAAGCGCTACACCGAGGACAAGTTCATGTTCCACGTGCCCATCCAGATCAACTACGGCGCCGAGAACCTGGGCCCCGTGAAGCTGAACAACCGCATCAACGAGAACATCGACGGCTTCACCCACATCATCGGCATCGACCGCGGCGAGCGCCACCTGGTGTACATCAGCGTGGTGGACGTGAAGACCGGCAAGATCGTGGAGCAGAAGCACCTGGACGAGATCGTGAACATCGACAGCAAGGGCAAGAAGCACTGCACCCCCTACCTGCAGAAGCTGGACGAGCGCAGCAAGACCCGCGACCAGGAGCGCAAGAGCTGGGAGGCCATCGAGACCATCAAGGAGCTGAAGGACGGCTACATCAGCCAGGTGGTGAACGAGATCTGCACCCTGCAGCAGAAGTACAACGCCCTGATCGTGATGGAGAACCTGAACCTGGGCTTCAAGCGCAGCCGCTTCAAGGTGGAGAAGCAGATCTACCAGAAGTTCGAGACCGCCCTGATCAAGAAGTTCAACTACATCATCGACAAGAAGGACAACAGCACCTACCTGCACGGCCTGCAGCTGGCCAACCCCATCCAGACCCTGAACAGCATCGGCAAGCAGAGCGGCATCATCTTCTACATCCCCGCCTGGAACACCAGCAAGATCGACCCCACCACCGGCTTCGTGAACCTGCTGTACGGCGCCGACCTGCGCTACACCAACAAGGAGCAGGCCGAGGCCTTCATCAACAAGCTGGACAAGATCTACTTCGAGGACGGCGTGTTCAAGTTCGACATCGACTTCAAGAAGTGGAACCAGCGCTACGCCAAGAGCTGCACCAAGTGGACCCTGACCAGCTACGGCACCCGCGTGGAGACCAAGCGCGACGTGATCAAGAACAACATGTGGTGCAGCAACGAGATCGACCTGACCGCCGAGTTCGAGAAGATCCTGAACAAGCGCGACGGCAGCCTGAAGACCTGCGACGTGGAGACCTACAAGCGCTTCCTGTACCTGTTCAAGCTGCTGCTGCAGATCCGCAACAGCATCACCGGCACCGACACCGACTACATGATCAGCCCCGTGATCGCCGCCGACGGCCAGCAGTTCGACAGCCGCGTGGTGGGCATGAGCCTGCCCAACGGCCTGCCCAAGGACGCCGACGCCAACGGCGCCTACAACATCGCCCGCAAGGGCCTGATGGCCGTGCACAACATCAAGGCCGGCTTCAAGAAGCCCTTCGAGATCAGCAACGAGGAGTACCTGGAGTACCTGCAGAAGaaaaggccggcggccacgaaaaaggccggccaggcaaaaaagaaaaag | | |
| The position of the sequence of EbCas12a at GenBank  SAAR01000186 (from 1416 to 4892) | ATGAGTAAGTTTCAAAATCTTTATACCATTAATAAGACACTTCGTTTTGGGTTGAAACCTTTTGGTAAAACCTTAGAAAATTTTAATAAAACGAATTTGTTACAACTGGATGAATATAAAGCTAAGCATAGAAAAGAAGTACAAAGGTTGTTTGATGAAAATTTTAAACAACTGATTGAAGAACGATTACGAGGTCTTTCTTTAGATACACAAGCTCTTGAAGAAGCTTTTGATATCAATAAACGTGATGCTGCATTAATATCTCTAAAAAAACAAGTGACAGGTATTTGCTATGATTCTGAAATGAAAAAAACATATTTACAGGCAGATAAGCATTTCCAAAAACTCTTAGCGGCAGGTCCTAATCAAGCTATGGTATGTACATATGATAAGTTTAGTACTTATTTTGTGAATTTCTTTGACATTCGTACGCATATTTTTAAGGGAGATACATCAGGGTCTATTGCTTACAGATTAATTGATGAAAATTTAACGATTTTTAAAAAGAATGTAGATAAGATTGCTAAATTACCAGTAGGTTTAAAAGATGAAGTTGAAGAATTAGCGGACATAGAGTCTTTACAAAGCTATAATTCCTATCTTACCCAATCGGGTATTACTGAATATAATGAGCTTTTAGGAGGTATTGCTTATGAAGATGGTACAAAACTACAAGGGATTAATGAAAAAATCAACTTATACGGGCAAAAGAATAAATTAAAATTACCTCGACTAGAATCGCTGTATAAAATGATTTTATCAGATAGAGAAACGCAGTCCTTTGTTTTATCTATCATTGAAAACGATGCAGAGTTGATTGGACAAATTTCAACTTTACTAGAAGATGTATTATTATCTAAAACATTAGCACTTTCTGATGTAGATGGAGTATTTATTAAATATACACAACTAGGCAATTTGCCAGGCGTACCATATACAGTGATTAACAGCAAAATTAATGAAGCTTTTGATGCTACTTATACAGGTAAAAAAGAAGGGGAAAAATACAAAGTTACAAAGAAGAAAACAATTGAAAAAGATGTATACTCGTTAAGTAAAATAGAGAAGCTGTTCCAGGACAGTGACATCAATGTTTCAGAGGCGTTGAAAAATAAGTATGGAGTGCTTATTGCTTCTTATGAAGAAGCAAAGGGGCTGTTTAATAGTATAGACTGGACAGAAATTAAAAATATAAAACAGTCTGGTCACACAATTATCATCAAAGATGTTTTAGACGCACTAAAAAATATCCAATTCTTCTATAATTTATTTGATGTTGTAGAAGAAAATTTAAATCCTTCCATCGAGTTTTATAATGAGCTAAGCTTGAATAAAAATCAGCTAGGGAATGAATTTAATAGTACGTACAATAAAGCAAGAAACTACTTAACTAAAAAAGAATACTCTGAGGAGAAATTCAAACTTAACTTTGACTCGCCAACATTAGCAGACGGCTGGGATGTAAATAAAGAAACGGCTAACCTTACTATATTATTGAGGCGTTTTAATAGTGAAAGAAATAATTATGATTATTTCTTGGGCGTTTGGAAAAAAGCTGTACCAAGTAGAGAAAAGAATCTAATCATTAATGCTGATGGTGAATTTGAAAAAATGGATTATAAACTATATCCTGATCCATCAAAAATGCTACCAAAGCAATTTGTTAGTGCTCAATCTTGGTTTGACAAATACCCAGCATCACCAGAGTTTATGGGAAAATATGAGGCAGGTCTTCATAAAAAGGGAAATAATTTTGATATAGAATTCTTGCATGAACTAATTAATCGCTATAAACATGGTTTAAAACATCATGAAAATAAATATGAGGAAACTTTTGATTTTGAATTAAAGGAAACAGAGGAATACTCTGAATATTCAGAATTTATTCAGGATGTAAGTAAAAGTAATTATAAAGTTAAATTTAATCATGTAGCAGGAGTAGAGGAATTAGTTGAAGAAGGAAAGTTATATTTATTCCAAATATGGTCTAAAGATTTCTCTACTTTCTCTAAAGGCACTAAAAATTTAAATACTATCTATTTTGAAAGTTTATTTTCTGAAGAAAATCTAGAAAAAAGGATTTTTAAGCTATCTGGTGGTGCAGAATTATTCTACAGACCTAAATCCTTAACATATACTAAAGAGCTTATGGAAAAAGGGCATCATTATAATGAGTTGAAAGATAGTTTCAACTATCCAATTATTAAGGATAAACGGTATACAGAAGATAAGTTTATGTTCCATGTACCAATCCAAATTAACTATGGTGCAGAAAACTTAGGTCCAGTTAAACTAAATAACCGAATAAATGAAAATATAGATGGATTTACACATATTATTGGTATAGACAGAGGGGAGCGCCATCTTGTTTATATCTCTGTAGTAGATGTGAAAACAGGAAAAATTGTAGAACAAAAACATTTAGATGAAATTGTTAATATTGATTCGAAAGGGAAGAAACATTGTACTCCGTACTTACAAAAACTAGATGAAAGAAGTAAAACTAGAGACCAAGAACGTAAATCTTGGGAAGCTATTGAAACGATTAAAGAACTAAAAGATGGCTATATCTCACAAGTTGTAAATGAGATTTGTACACTGCAACAGAAATATAATGCACTCATTGTTATGGAGAACCTCAATCTAGGGTTTAAACGGTCACGTTTTAAAGTGGAAAAACAAATTTATCAAAAATTTGAAACAGCACTAATCAAGAAGTTTAACTATATTATTGATAAGAAAGATAATTCTACCTACTTACATGGTTTACAATTGGCCAATCCTATTCAAACTCTAAACAGTATAGGGAAGCAGTCGGGTATTATTTTCTACATTCCTGCATGGAATACATCTAAAATTGATCCAACAACAGGGTTTGTAAATTTACTTTATGGAGCAGATTTGAGATACACCAATAAAGAGCAAGCAGAAGCTTTCATTAATAAGCTAGATAAAATATATTTTGAGGATGGTGTATTCAAATTTGATATTGACTTCAAGAAATGGAACCAGCGATATGCTAAATCTTGTACAAAATGGACTCTAACTAGTTATGGTACTCGCGTTGAGACAAAACGAGATGTAATTAAAAATAATATGTGGTGCTCTAATGAAATTGATTTGACTGCAGAGTTTGAAAAAATACTTAATAAAAGAGATGGAAGCTTAAAAACTTGTGATGTAGAAACATATAAGCGATTCTTGTACTTGTTTAAGCTATTGCTCCAAATTCGTAACTCAATTACTGGCACGGATACTGACTATATGATTTCACCAGTTATAGCTGCTGATGGACAACAATTTGATTCAAGGGTTGTAGGGATGTCTTTGCCTAATGGGCTGCCTAAAGATGCTGATGCTAATGGTGCGTATAATATTGCACGAAAAGGACTGATGGCGGTGCATAATATTAAAGCAGGCTTTAAAAAACCATTTGAAATATCTAACGAAGAGTACTTAGAATATCTTCAAAAGTAA | | |
