# Supplementary material for: Engineering of a compact, high-fidelity EbCas12a variant that can be packaged with its crRNA into an all-in-one AAV vector delivery system
Source: PLoS Biol. 2024 May 30;22(5):e3002619. doi: 10.1371/journal.pbio.3002619 (PMC11139299; doi:10.1371/journal.pbio.3002619)

Extended Data Fig. 1: Uncropped gels for Fig 1C.

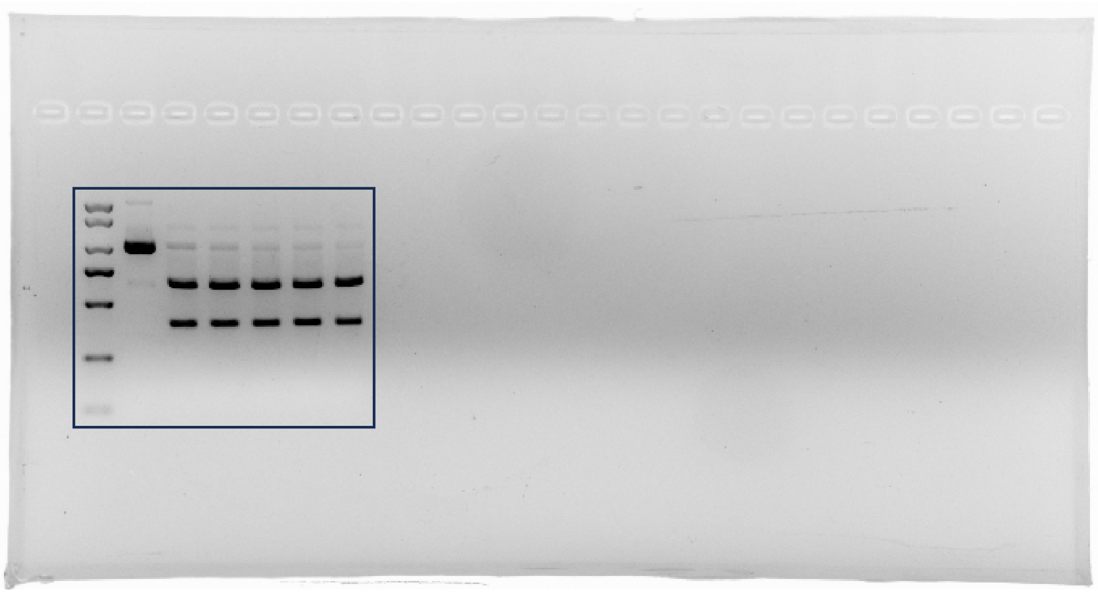

Extended Data Fig. 2: Uncropped gels for Fig 2C.

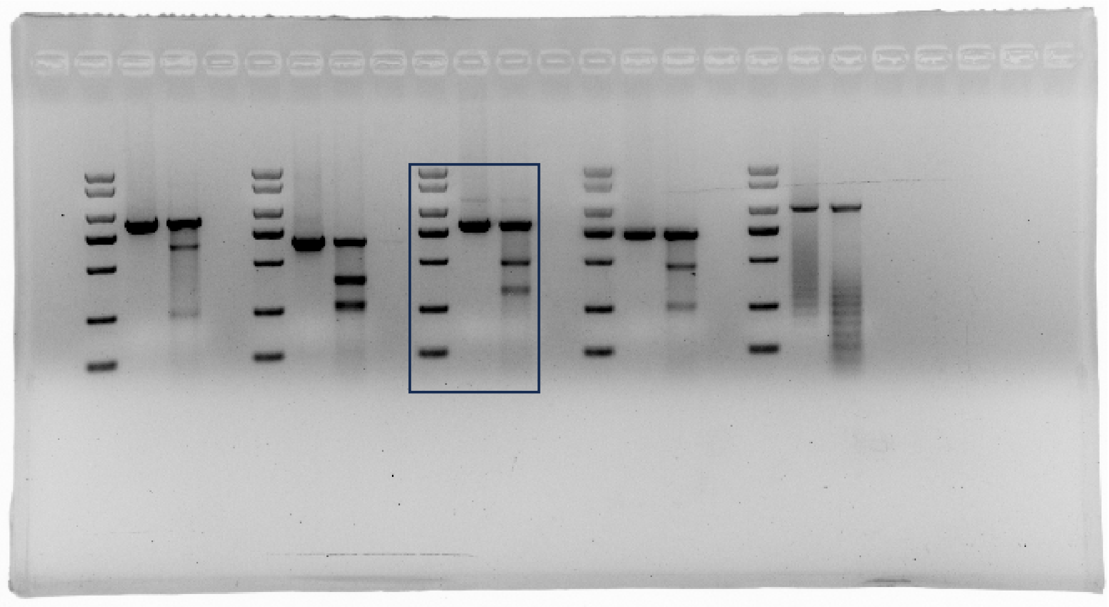

Extended Data Fig. 3: Uncropped gels for Fig 2E.

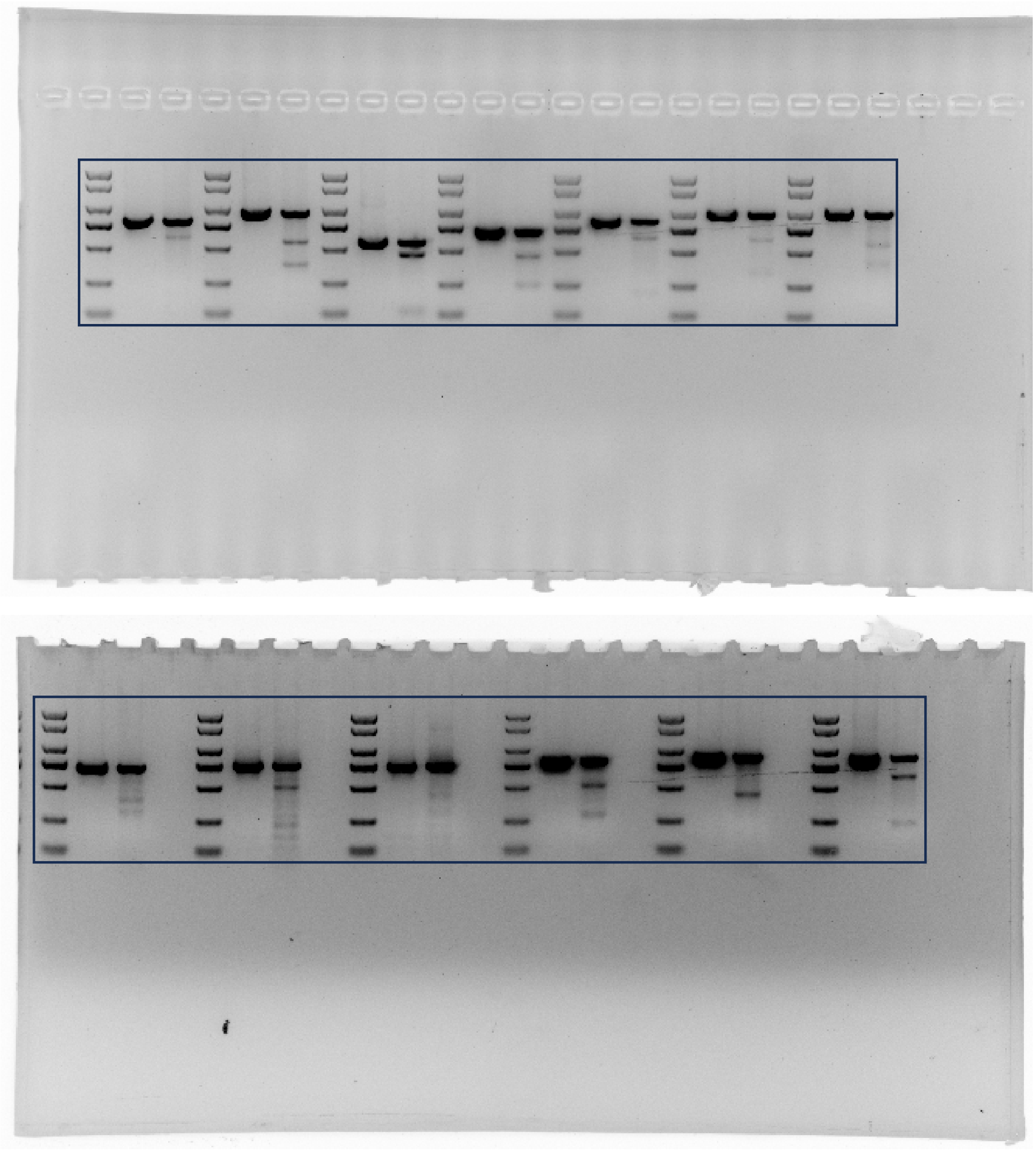

Extended Data Fig. 4: Uncropped gels for Fig 4B.

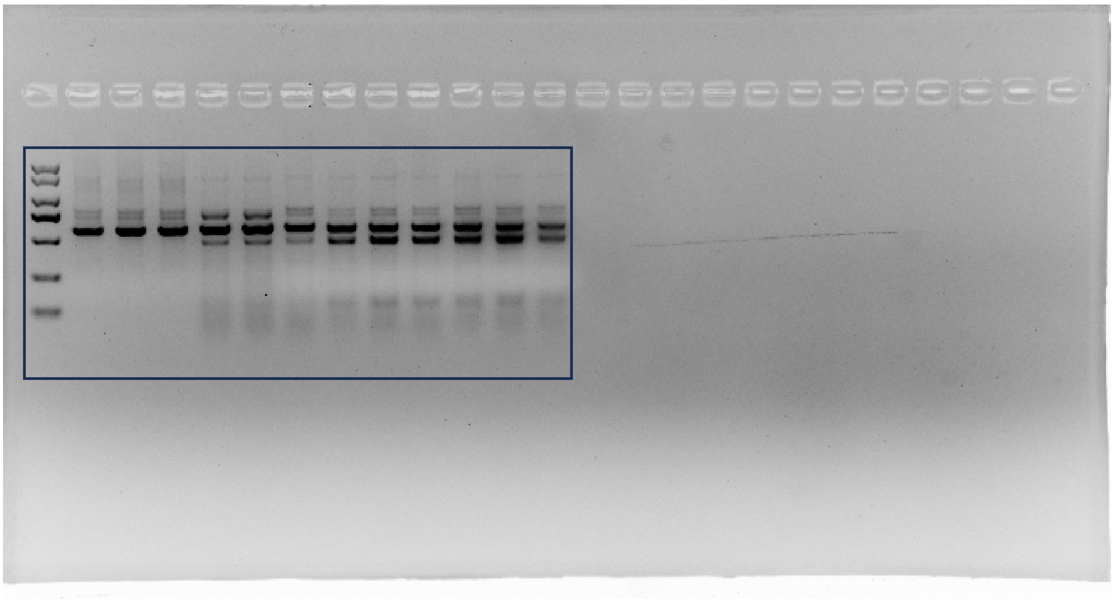

Extended Data Fig. 5: Uncropped gels for Fig 4C.

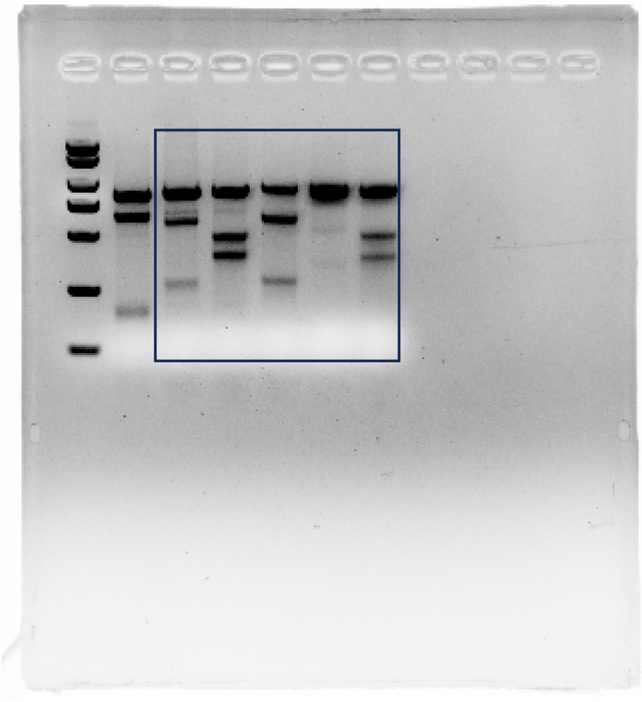

Extended Data Fig. 6: Uncropped gels for Fig S1B.

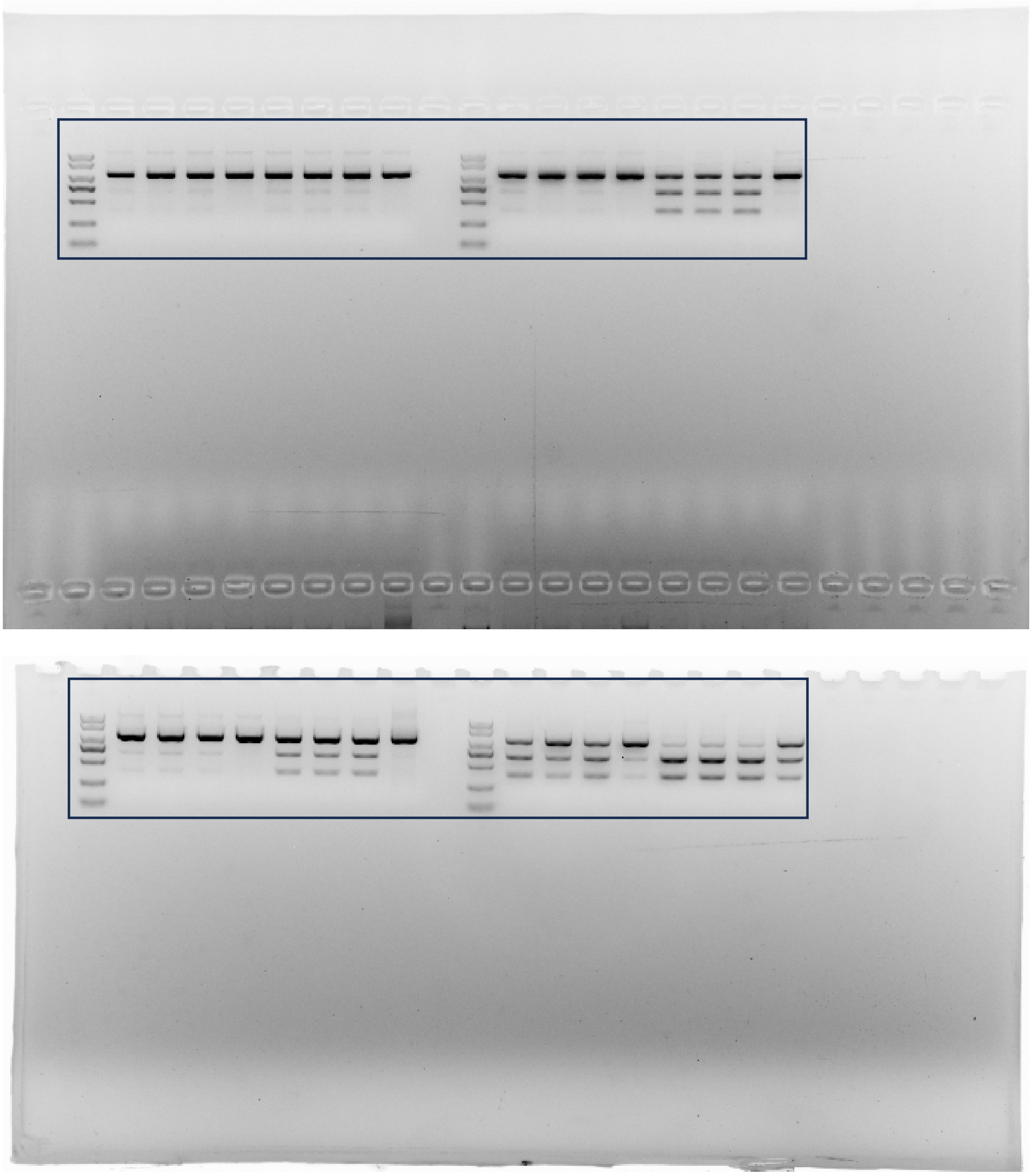

Extended Data Fig. 7: Uncropped gels for Fig S3.

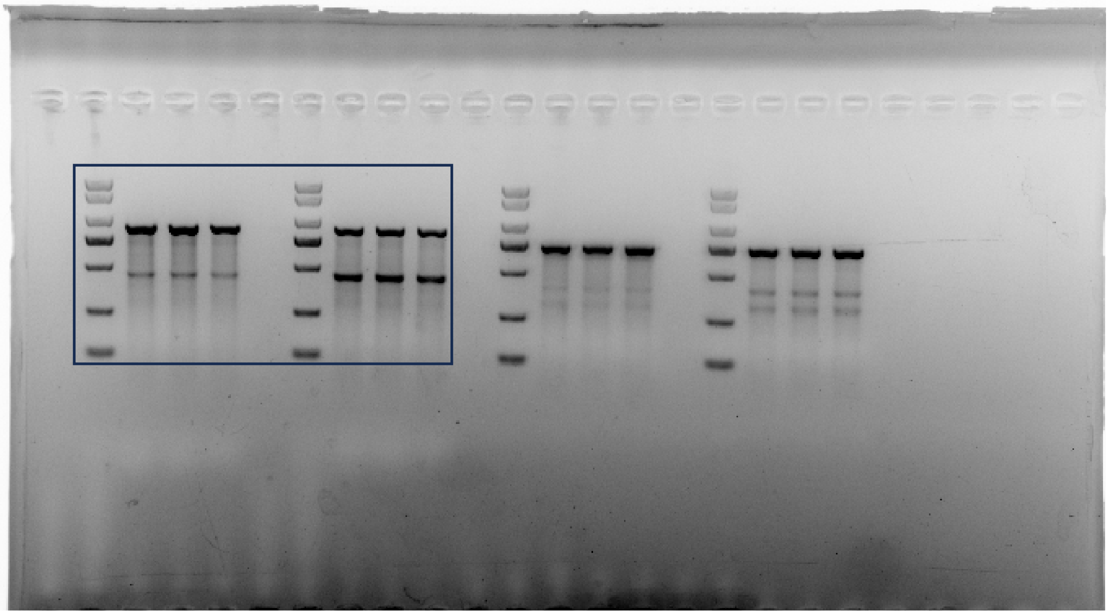

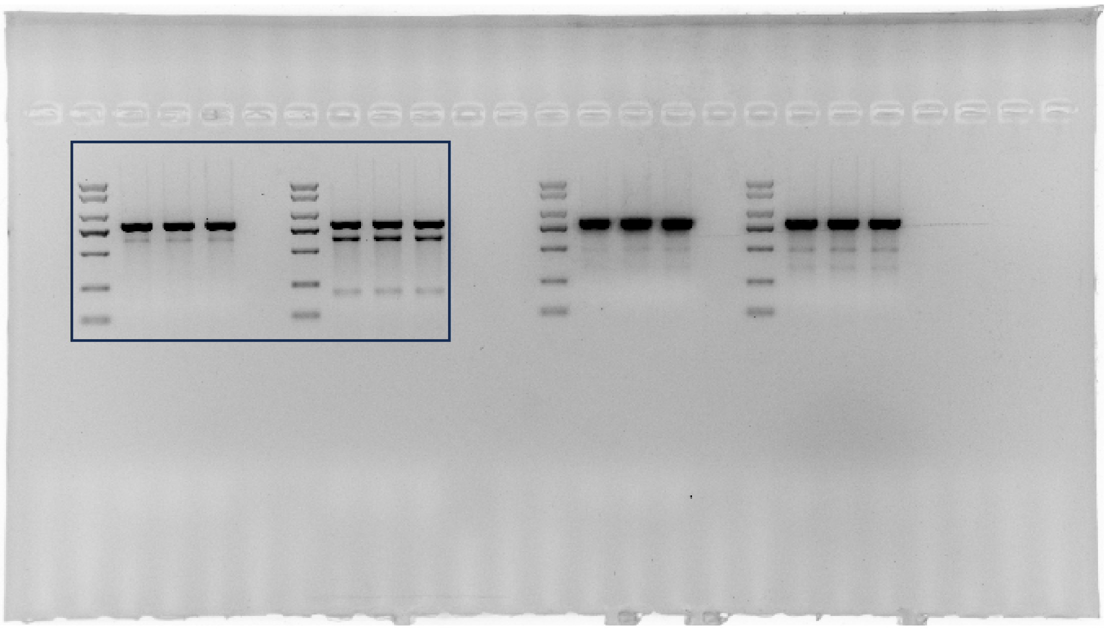

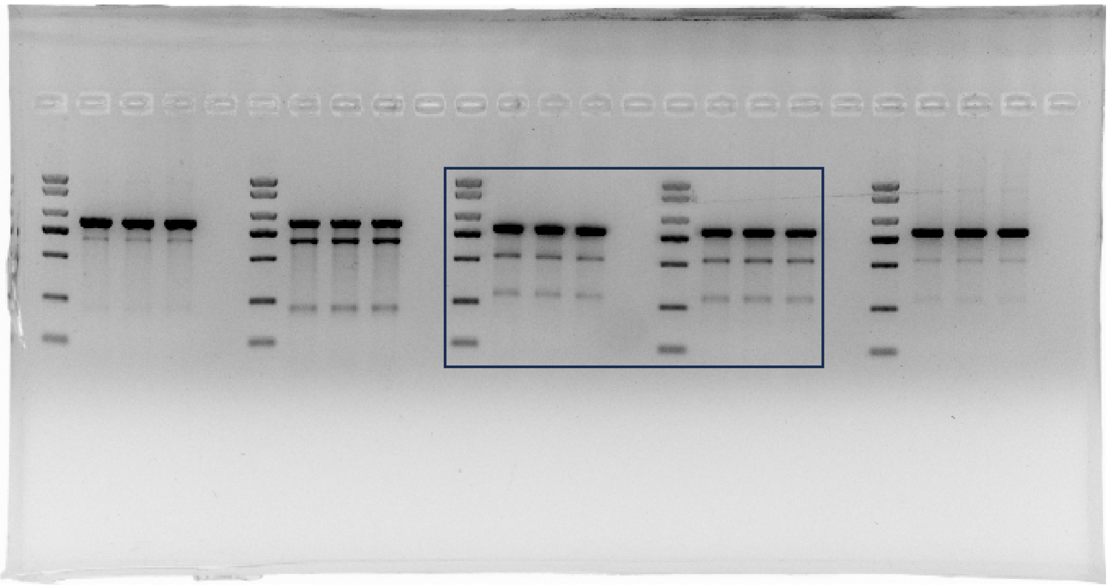

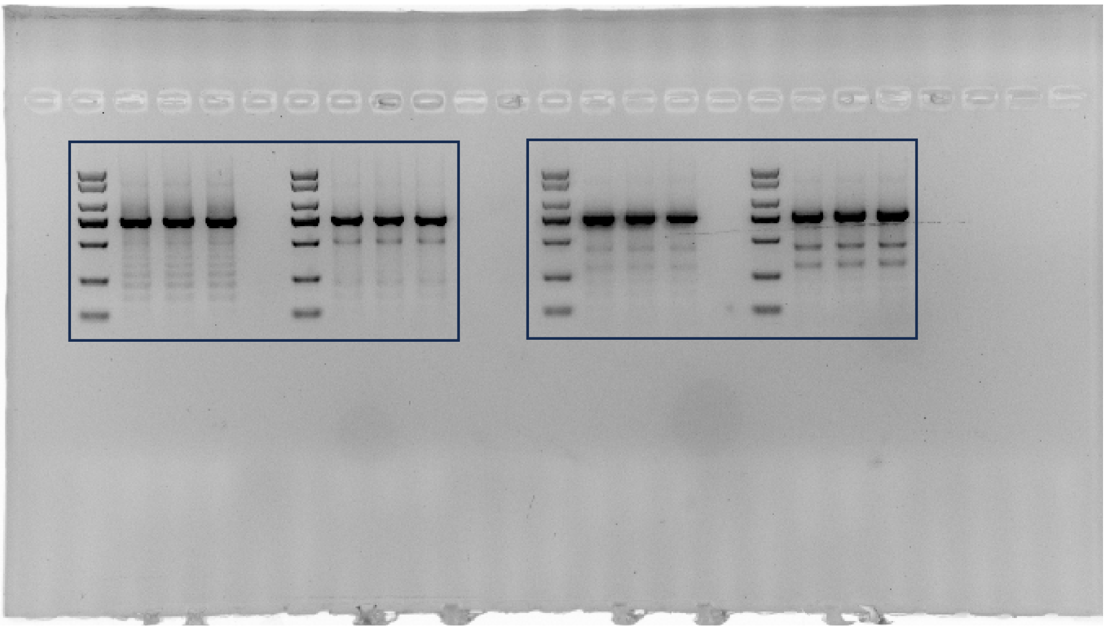

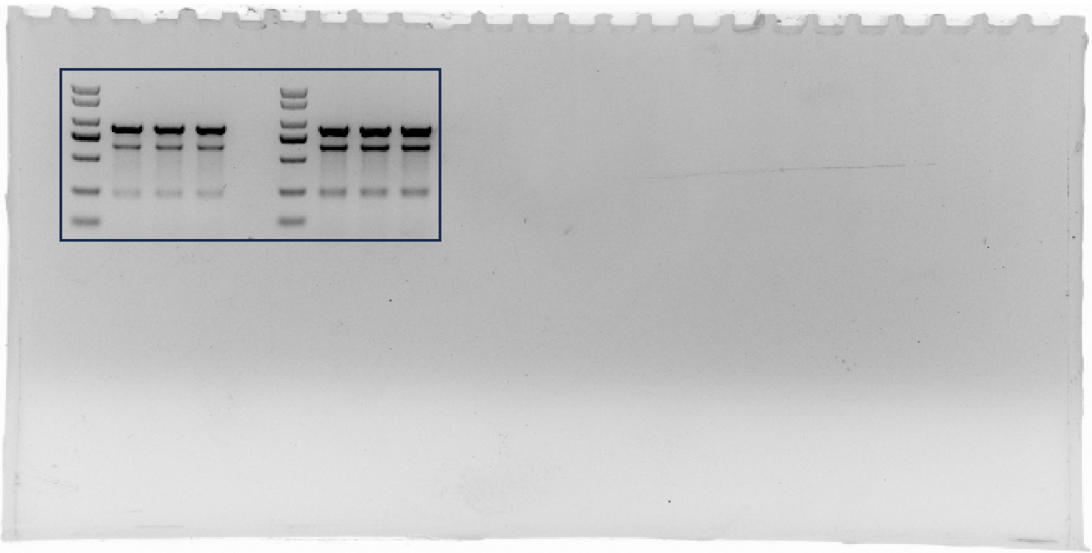

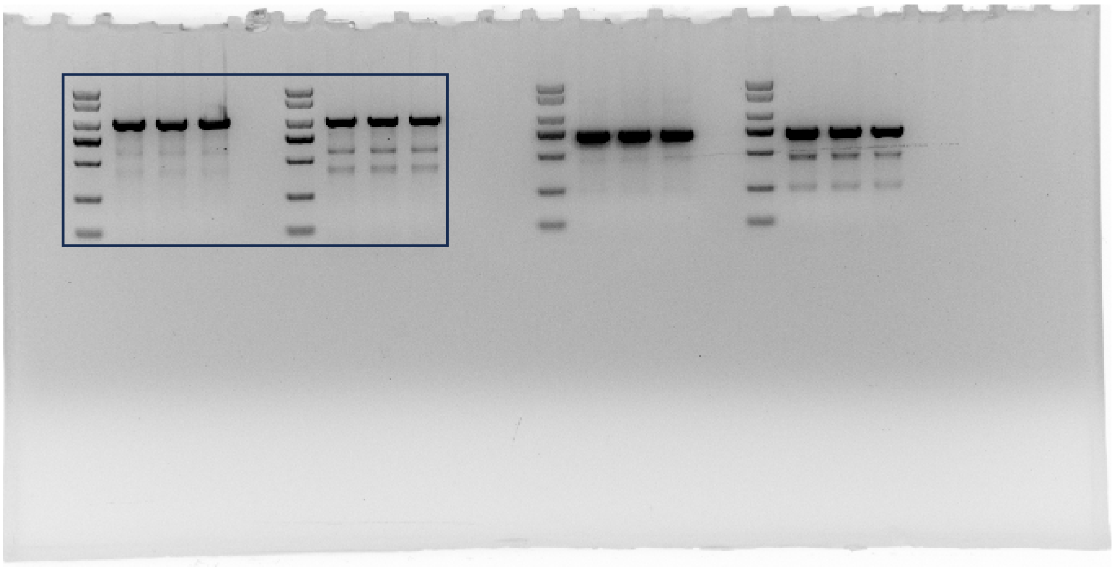

Extended Data Fig. 8: Uncropped gels for Fig S7A.

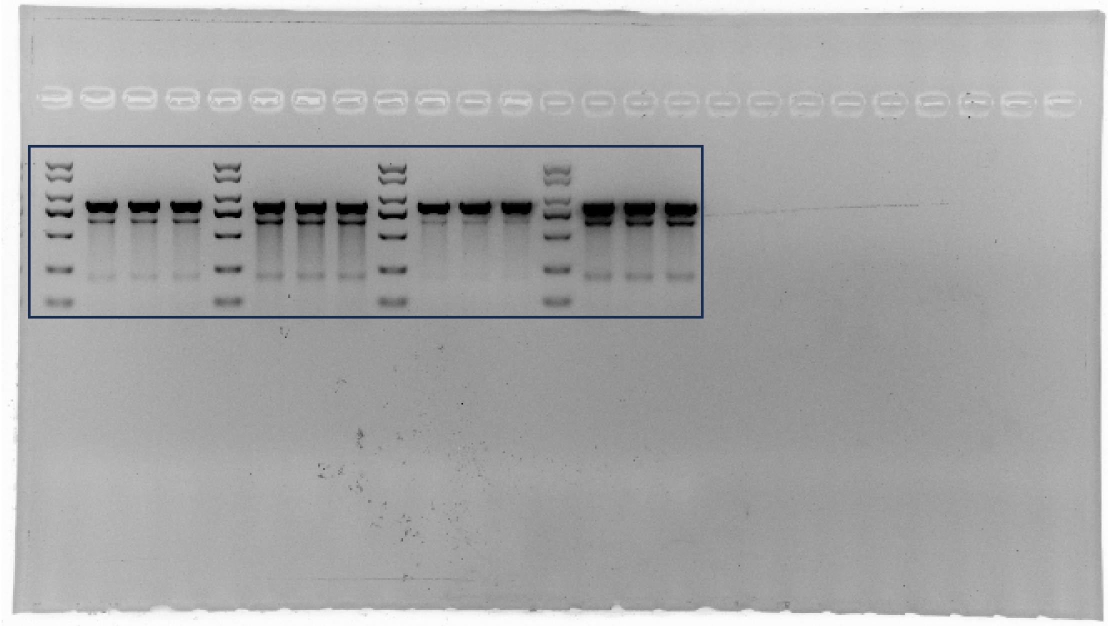

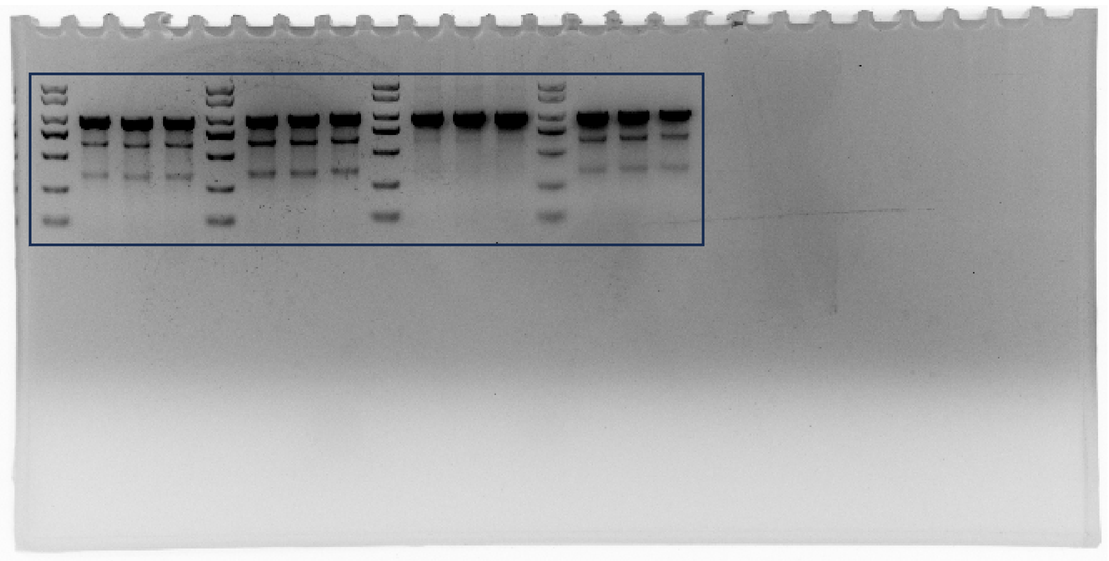

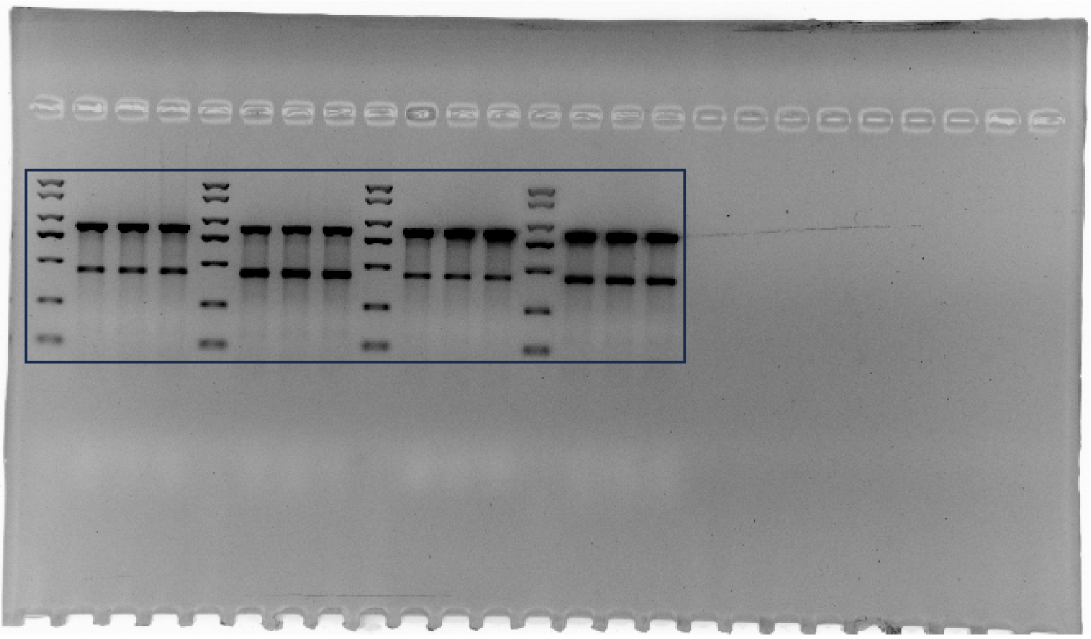

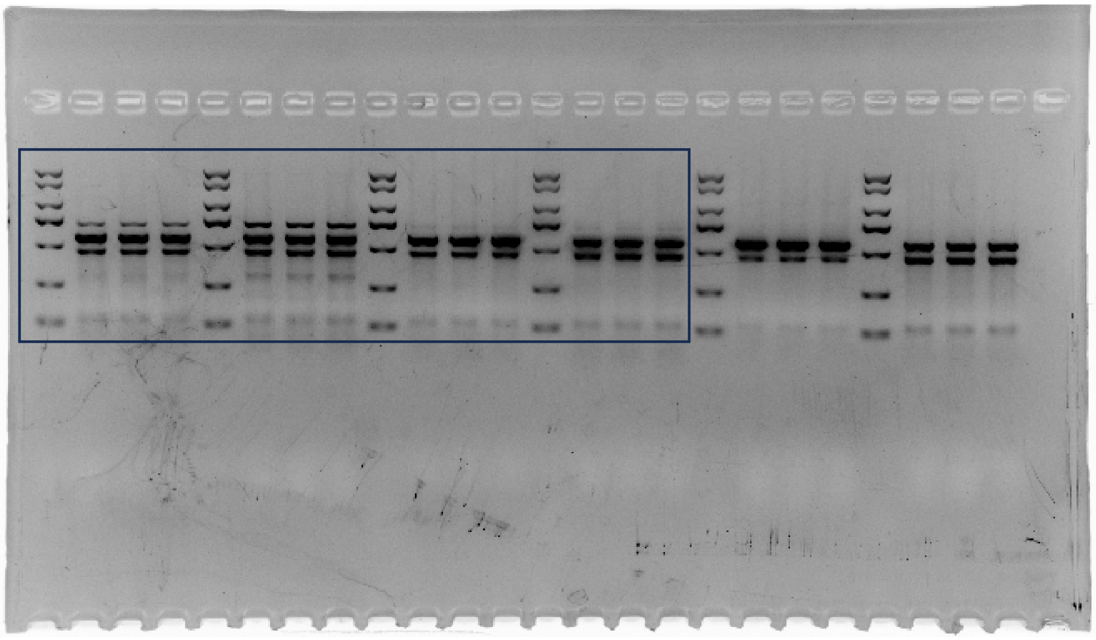

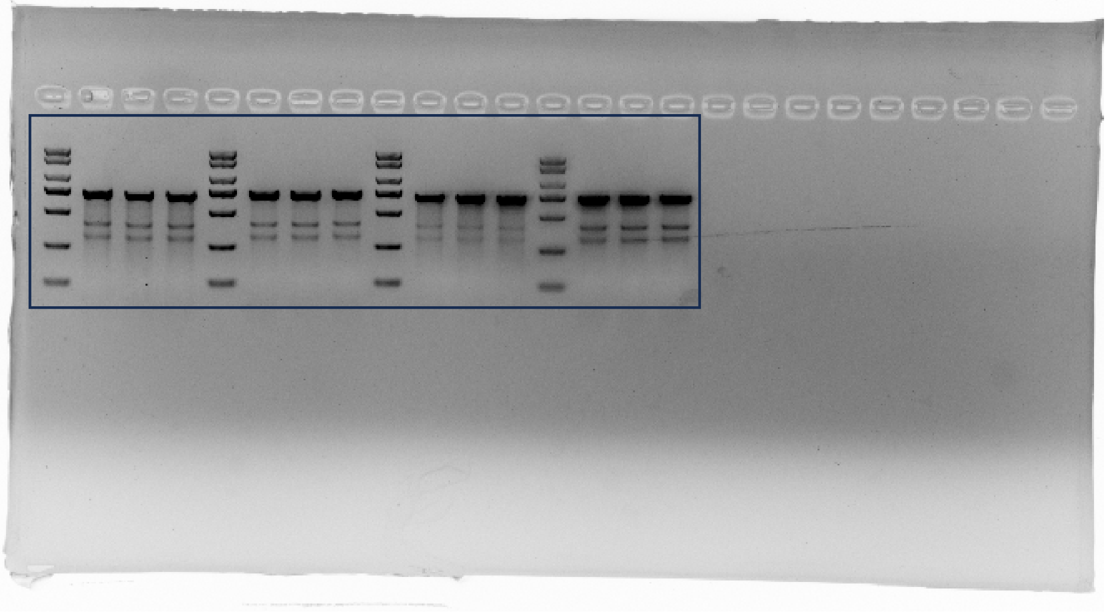

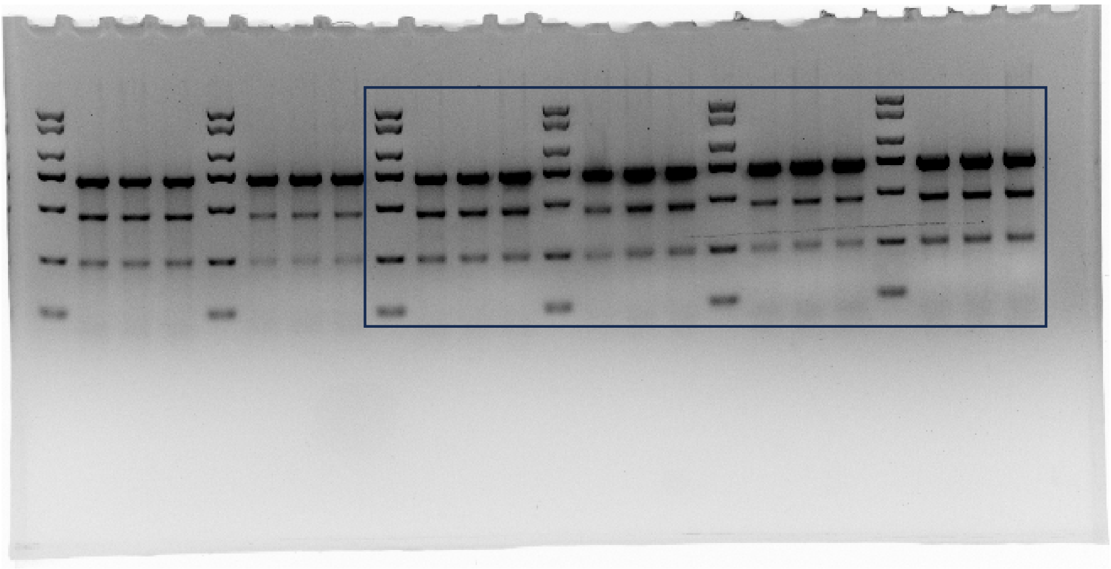

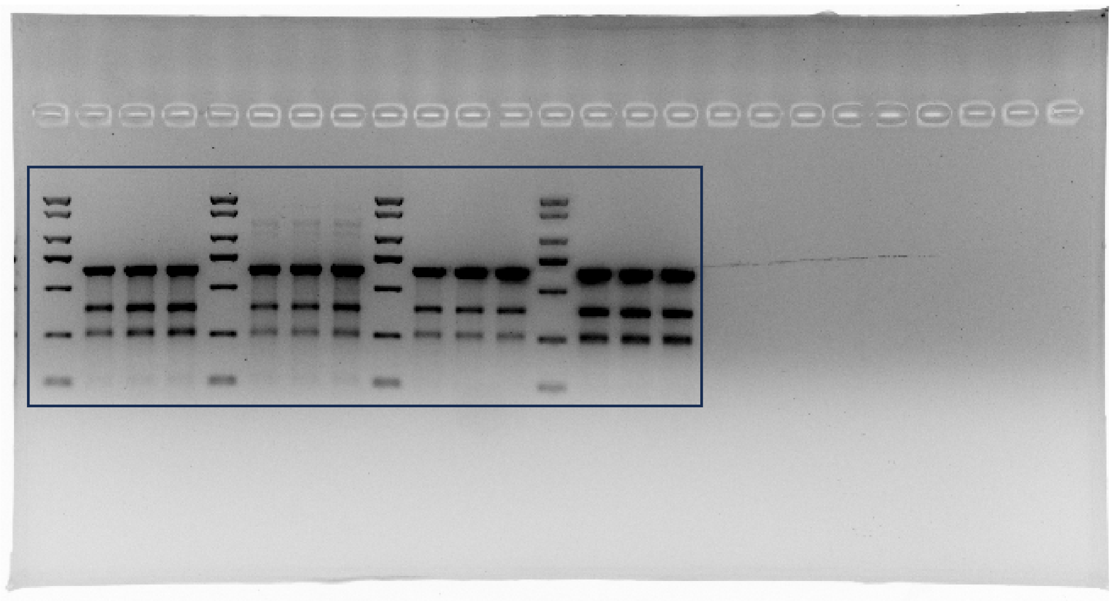

Extended Data Fig. 9: Uncropped gels for Fig S8A.

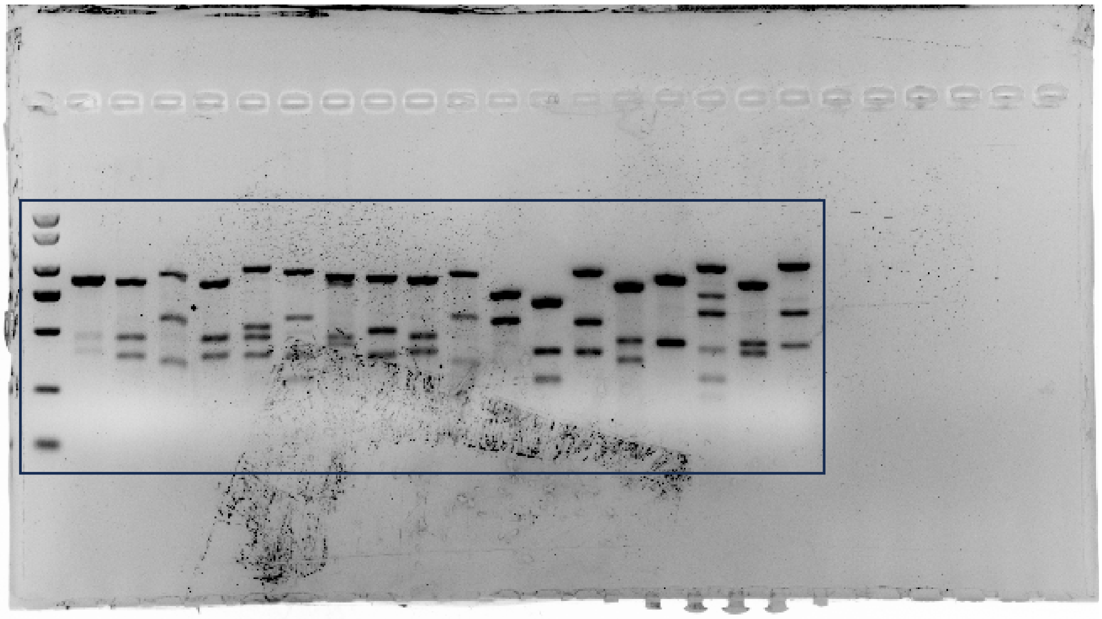

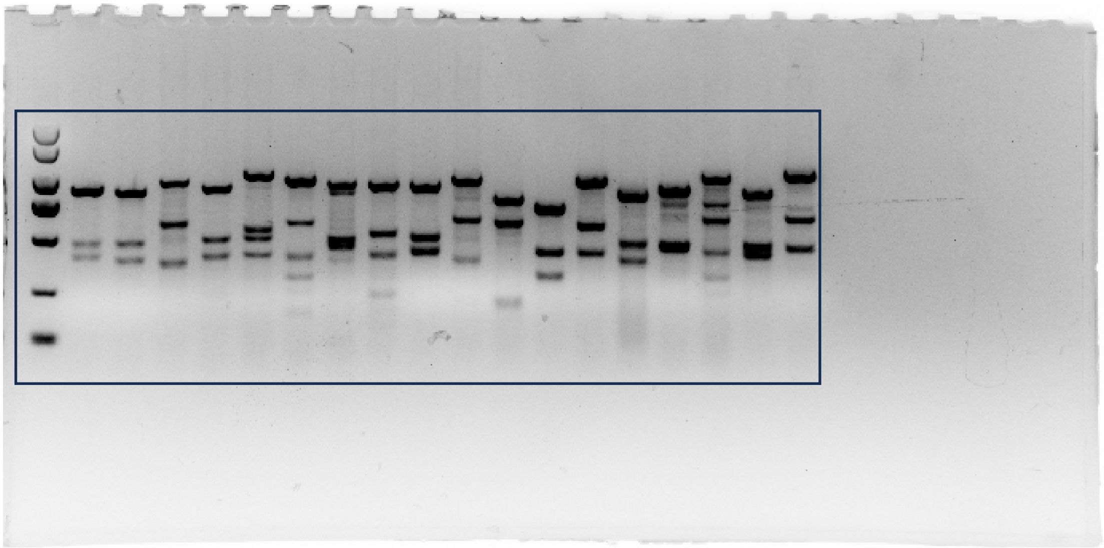

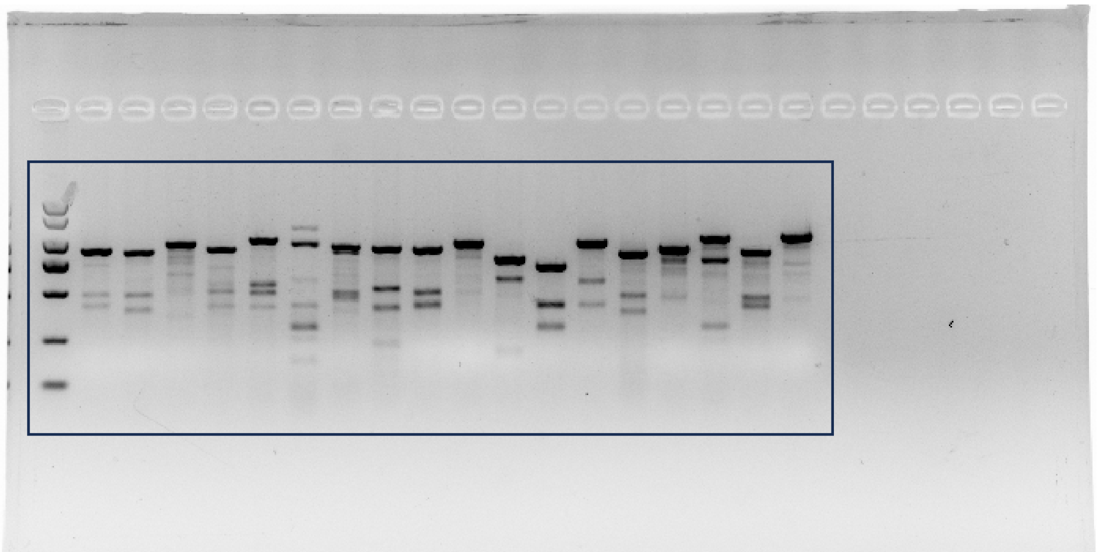

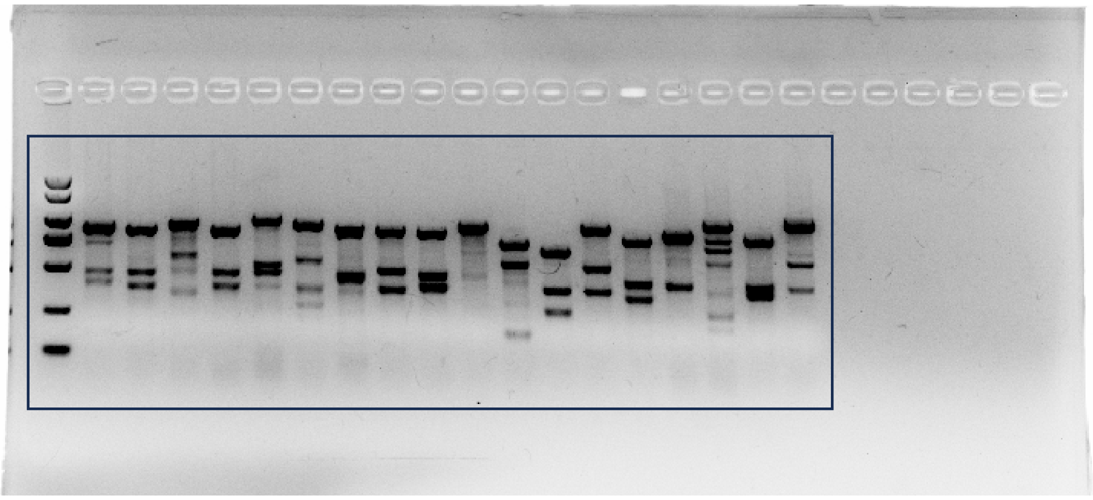

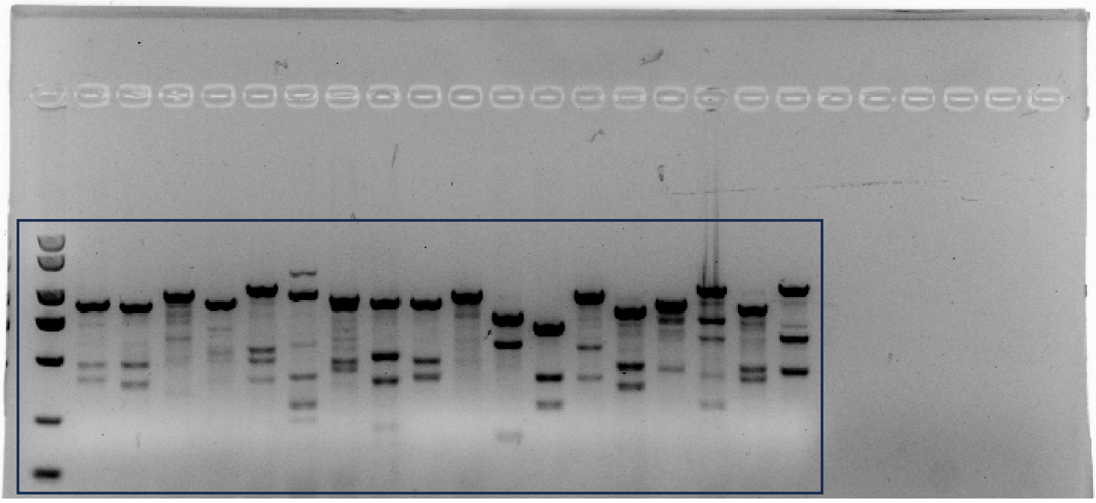

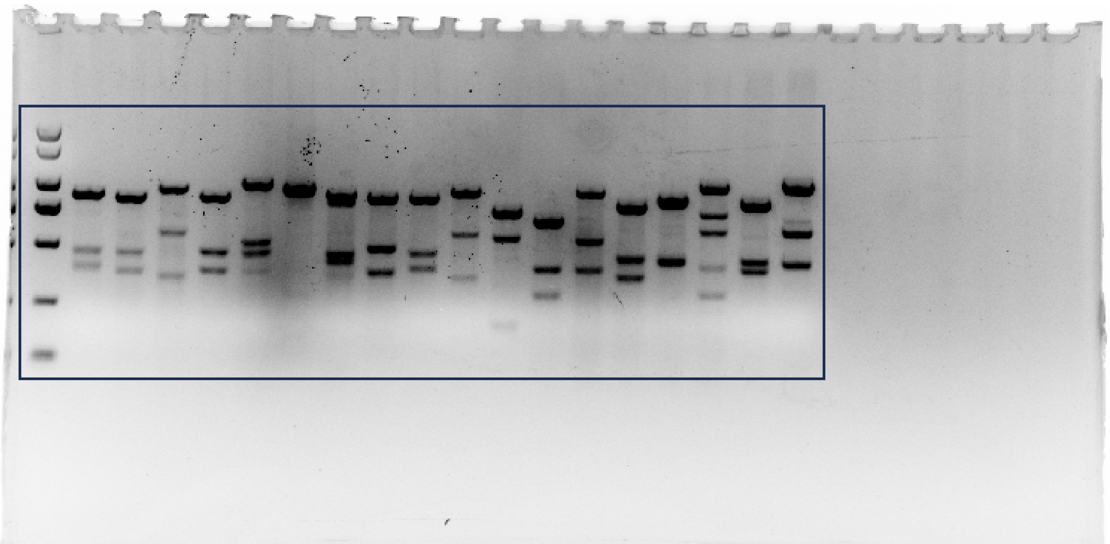

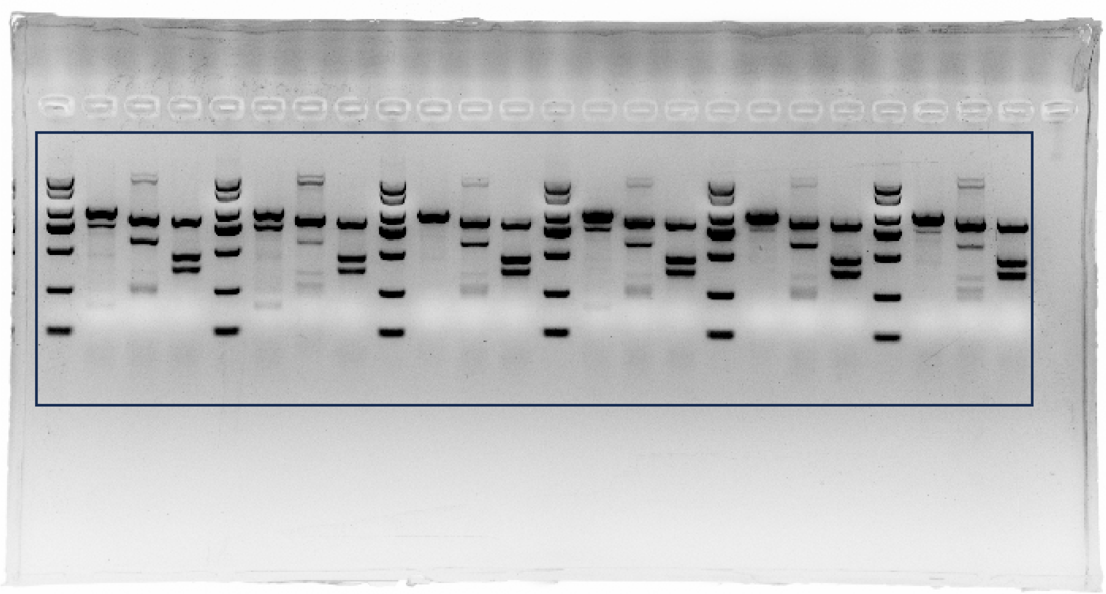

Extended Data Fig. 10: Uncropped gels for Fig S9A.

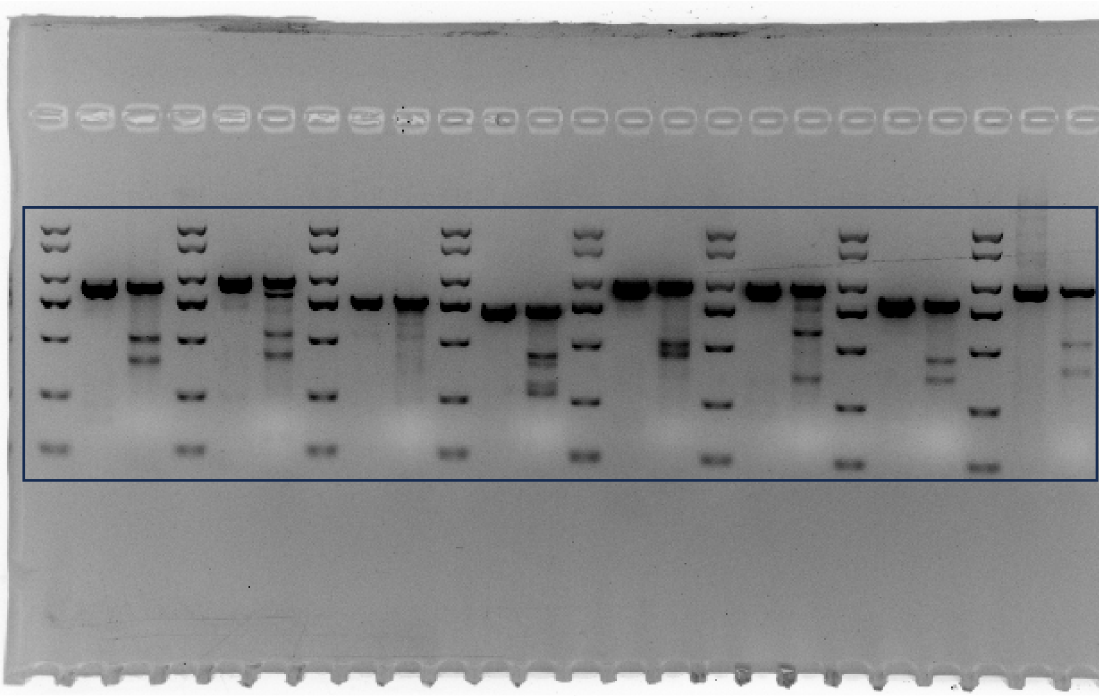

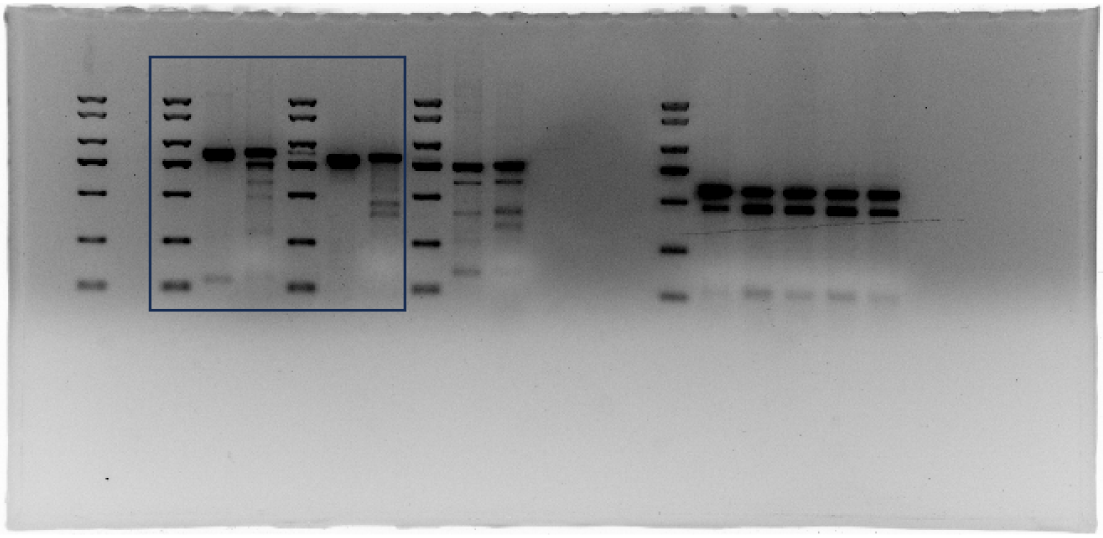

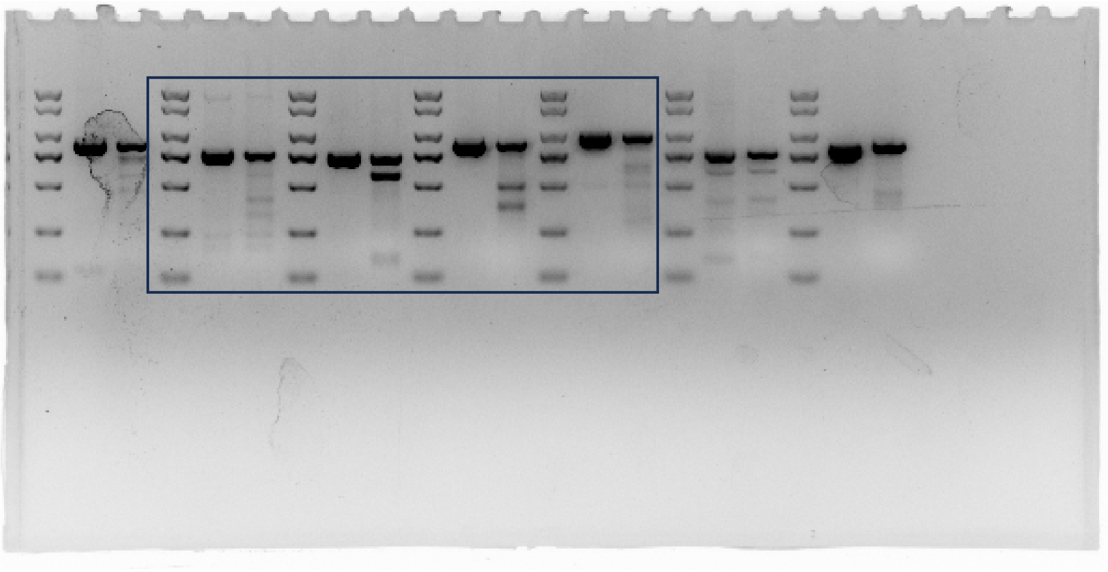

Extended Data Fig. 11: Uncropped gels for Fig S13A.

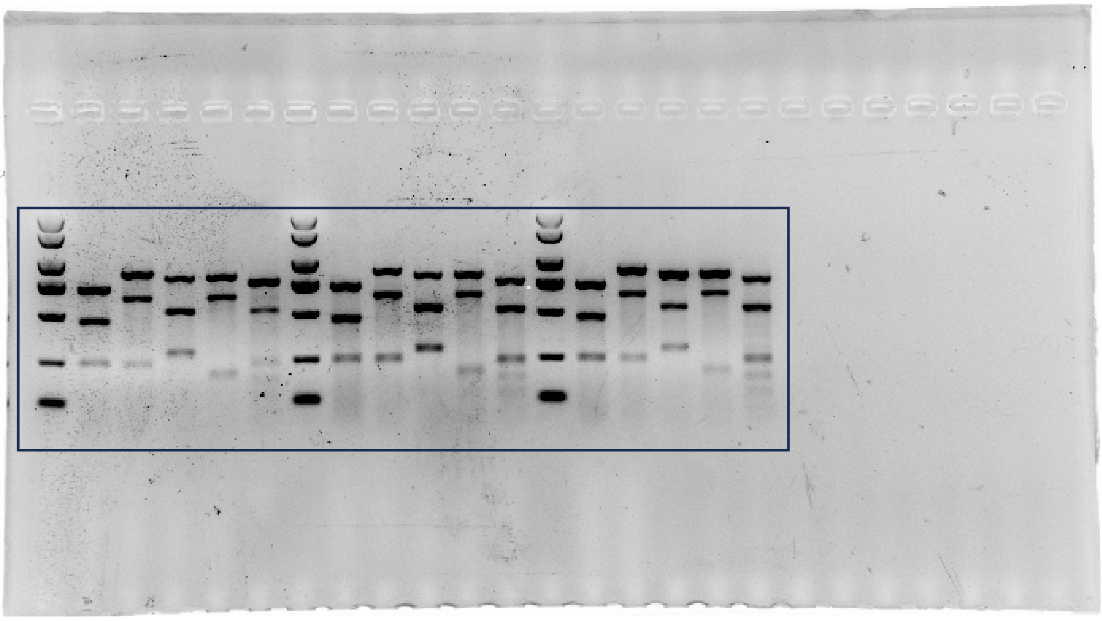

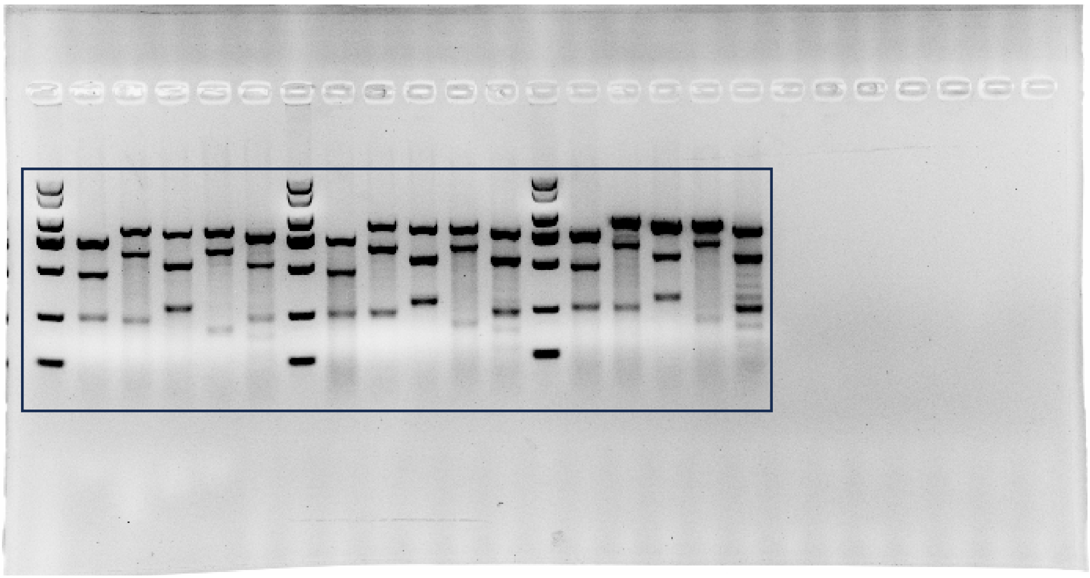

Extended Data Fig. 12: Uncropped gels for Fig S14D.

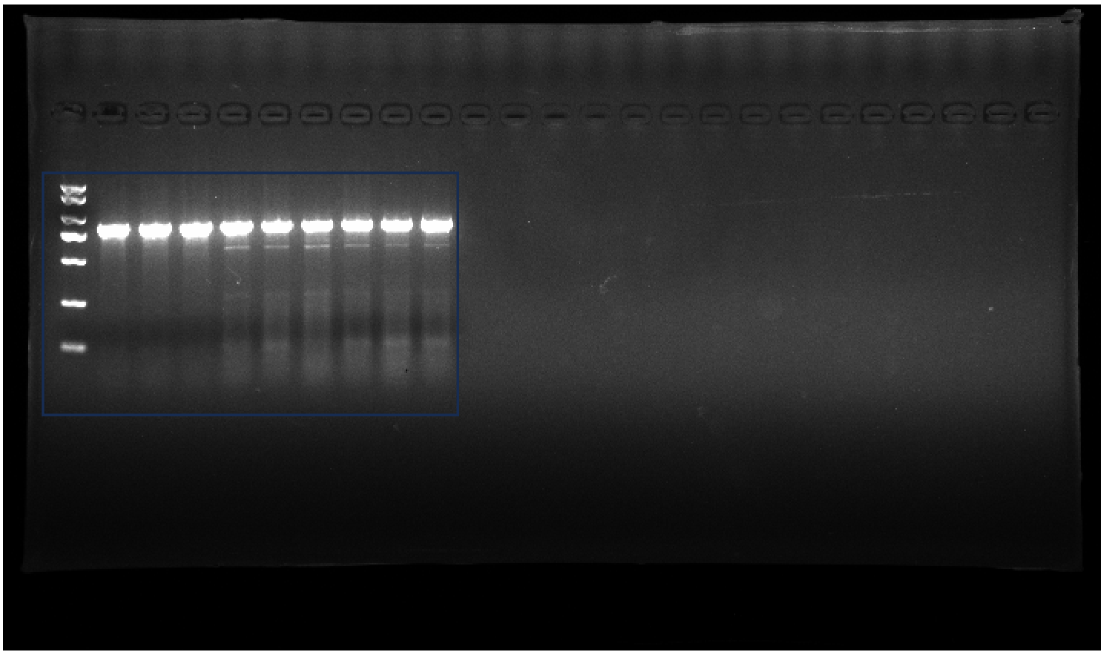

Supplement: S1 Raw Images — Abbreviations: PAM, protospacer adjacent motif; CRISPR-Cas, Clustered Regularly-Interspaced Short Palindromic Repeats and CRISPR-Associated proteins; EbCas12a, a Cas12a ortholog from a bacterial species of the class Erysipelotrichia; AsCas12a, Acidaminococcus sp. Cas12a; LbCas12a, Lachnospiraceae bacterium. Cas12a; RNP, ribonucleoprotein; SpCas9, Streptococcus pyogenes. Cas9; SaCas9, Staphylococcus aureus. Cas9; EGFP, enhanced green fluorescence protein; GUIDE-seq, genome-wide unbiased identification of double-stranded breaks enabled by sequencing; enEbCas12a, enhanced EbCas12a variant; enAsHF1, enhanced Acidaminococcus sp. Cas12a variant; WT, wild type; GCs, genomic copies. (PDF) [file pbio.3002619.s017.pdf]
