# Supplementary material for: Engineering of a compact, high-fidelity EbCas12a variant that can be packaged with its crRNA into an all-in-one AAV vector delivery system
Source: PLoS Biol. 2024 May 30;22(5):e3002619. doi: 10.1371/journal.pbio.3002619 (PMC11139299; doi:10.1371/journal.pbio.3002619)
Supplement: Attachment — Submitted filename: Response to Reviews.docx [file pbio.3002619.s018.docx]

Dear Editor and Reviewers:

Thank you very much for the letter and the reviewers' comments on our manuscript entitled "The compact high-fidelity EbCas12a enables all-in-one AAV delivery system" (ID: PBIOLOGY-D-23-01942R1). These comments are all very valuable and helpful in revising and improving our paper. We have read the comments very carefully and all of them have been addressed. Revised sections are track changes in the paper. Below we provide point-by-point responses to the reviewers' comments.

Reviewer comments:

Reviewer #1: Wang et al. demonstrated genome editing using a compact Cas12a, EbCas12a. They determined the TTTV PAM sequences of EbCas12a and developed engineered version of EbCas12a, enEbCas12a. Next, the activity and specificity of enEbCas12a were compared with compared with AsCas12a and LbCas12a. Lastly, the authors investigated a single AAV system with EbCas12a and crRNA in vitro and in vivo.

*1. In Figure 3E, the activity of enEbCas12a was compared only with wildtype AsCas12a and LbCas12a at seven target sites. To establish the utility of enEbCas12a, it would be necessary to measure and compare editing efficiency at a sufficient number of target sites where statistical significance can be demonstrated. I strongly recommend including the latest Cas12a variants, such as enAsCas12a (PMID = 30742127) or Lb2Cas12a (PMID = 33738137), for comparison.*

Response:

Thank you very much for the valuable advice, and we fully agree with the reviewer that it is necessary to measure and compare the editing efficiency with other Cas12a variants. We detected another 21 endogenous target sites including *Fgfr1*, *Flt3*, *Ntrk2*, *Tek*, *Axl*, *Rxra*, *Egln2*, *Nqo1*, *Alox5*, *Pde7a*, *Chrnb1*, *Pkm2*, *Drd1*, *Top2a*, *Kit*, *Pdgfra*, *Vegfr2*, *Src*, *Adra2a*, *Ret*, *RACK1* (**Response Fig. 1A)**. One day prior to transfection, approximately 1.2×10^5^ cells were seeded per well of a 24-well plate, and 500 ng Cas12a and 250 ng crRNA expression plasmids were transfected into the cells using Hieff Trans Liposomal Transfection Reagent. 48 hours after transfection, cells were harvested and lysed in 50 μL lysis buffer. For the T7E1 assay, 1 μL of cell lysis was used as a template to amplify the target genomic region. PCR products were purified and concentrations determined. 250 ng of purified PCR products were used to perform the T7E1 cleavage assay (**Response Fig. 1A)**. As shown in **Response Fig. 1B**, AsCas12a, enAsCas12aHF1, EbCas12a, enEbCas12a, Lb2Cas12a, LbCas12a possessed 31.1%, 38%, 15.7%, 32%, 23.2%, 33.4% of the average on-target cleavage efficiency. In addition, enEbCas12a significantly increased gene editing efficiency compared to EbCas12a WT. This figure has been added in the revision of **Fig. 3E and S8 Fig.**

**Response** **Figure 1:** Evaluation of the activity of AsCas12a, enAsCas12aHF1, EbCas12a, enEbCas12a, Lb2Cas12a, LbCas12a. **A**, Activity of Cas12a variants on targets with candidate PAM sequences assessed by T7 endonuclease I assay. **B**, Average activity of Cas12a variants on targets; summary of on-target modifications from **A**. Each dot represents one target site. Mean with SD is shown. **P < 0.005, ***P < 0.001 (one-way ANOVA).

*2. If possible, it would be beneficial to perform an analysis of the features of target sites where* *enEbCas12a exhibits higher efficiency compared to other Cas12a variants.*

Response:

The reviewer has made a good suggestion and we appreciate that very much. We summarized all target sequences used for enEbCas12a and performed an analysis of the target site characteristics (**Response Fig. 2A and 2B**). However, we did not find features of the target sites where enEbCas12a shows higher efficiency (**Response Fig. 2B**). This may be due to the fact that there were not enough target sequences available for analysis and therefore no useful sequence features could be obtained. We will follow up on this issue in future studies and summarize whether enEbCas12a has a preference for cleaving target sequences over thousands of target sequence analyses.

**Response** **Figure 2:** Analysis of the features of target sites. **A**, Summary of all target sequences used for enEbCas12a. **B**, Seqlogo of the target sequence feature.

*3. To comprehensively analyze off-target effects, it's crucial to ensure comparable on-target activities. In Figure 3F, we require a clear description of the on-target activities for AsCas12, LbCas12a, and enEbCas12a. It would be more accurate to measure indel frequencies at endogenous target sites rather than relying solely on Guide-seq read counts. Furthermore, to validate the identified off-target sites using Guide-seq, it's essential to confirm the presence of indels at these potential off-target sites through deep sequencing.*

Response:

Thank you very much for this good suggestion and we totally agree with that. To evaluate the on-target cleavage activity of AsCas12a, LbCas12a and enEbCas12a, we performed the T7E1 assay at the endogenous target sites *DNMT1*, *HEK site5*, *NLRC4*, *CLIC4*, *POLQ*. As shown in **Response Figure 3A** and 3**B**, AsCas12a, LbCas12a and enEbCas12a show comparable on-target activities. To validate the off-target sites identified by GUIDE-seq, we confirmed the occurrence of indels at the endogenous sites using deep sequencing. As shown in **Response Figure 3C**, AsCas12a, LbCas12a and enEbCas12a generated indels at these off-target sites detected by GUIDE-seq. This figure has been added in the revision of **S13 Fig**.

**Response** **Figure 3:** Deep sequencing validation the off-target sites identified by GUIDE-seq. **A**, Evaluation of the activity of AsCas12a, LbCas12a and enEbCas12a. Activity of Cas12a variants on targets assessed by T7 endonuclease I assay. **B**, Average activity of Cas12a variants on targets; summary of on-target modifications from A. **C**, Percent modification GUIDE-seq detected off-target sites with indel mutations for AsCas12a, LbCas12a and enEbCas12a.

*4. Although enEbCas12a is relatively small with a size of 1158 amino acids compared to the Cas12a family, there have been much smaller genome editing tools developed recently, such as Un1Cas12f (529 aa, PMID = 34475560) and AsCas12f (422 aa, PMID = 37400536). Among Cas9 variants, sRGN3.1 (1057 aa) and SlugCas9 (1054 aa) that showing robust activity (PMID = 37188955) also smaller than enEbCas12a . Therefore, an analysis and discussion of the advantages that enEbCas12a possesses compared to these smaller counterparts is needed.*

Response:

We are very grateful to your kind advice. We have added a section to analyze and discuss the advantages of the enEbCas12a compared to these smaller counterparts. "Changes Highlighted" can be found on lines 216-228 in the revised version. The text is as follows:

“Although a number of small Cas12f homologs[1, 2], such as AsCas12f1[2, 3], Un1Cas12f1[4, 5], CWCas12f1[6], OsCas12f1[7], and RhCas12f1[7], and a number of small Cas9 homologs[8], such as St1Cas9[8-11], Nm1Cas9[8, 12-14], SaCas9[9, 15-18], CjCas9[19, 20], and Nm2Cas9[21], SauriCas9[22], sRGN3.1[23] and SlugCas9[23, 24] have been identified and engineered to modify genomic DNA, Cas12a nucleases have been previously characterized as a generally low off-targeting family and have a unique ribonuclease activity to process their own crRNAs from longer precursors. In addition, a potentially important difference in biochemical behavior between Cas12a nucleases and Cas9 nucleases is the ability of Cas12a to cleave non-specifically single-stranded DNA (ssDNA) (*cis*-activity) following crRNA-guided target DNA binding and cleavage (*trans*-activity). Therefore, the distinct features may allow enEbCas12a to exhibit high fidelity and edit multiple genes simultaneously and potentially facilitate diverse virus detection.”

Reviewer #2: The authors have cloned and harnessed an enhanced variant of EbCas12a and demonstrate its compatibility with AAV vectors in cultured cells and in the mouse liver. The data are encouraging as their Cas12a version alleviates previous concerns about the size of Cas12a that have hampered packaging in and delivery by AAV.

Overall, this is an interesting article and a useful advance, thus I only have a few minor comments:

*1) Did the EbCas12a vector package well? What were the average titers as compared to other vectors in the authors' hands?*

Response:

Thank you very much for your favorable opinion, which recognized the interesting and useful advancement presented in the article. Yes, enEbCas12a vector packages efficiently. To compare average titers with other AAV-Cas12a-crRNA all-in-one systems, we constructed AAV-AsCas12a-crRNA, AAV-FnCas12a-crRNA vectors (**Response Fig. 4A**).

For virus production, HEK293T cells were grown in 150 mm plates in Dulbecco's modified Eagle's medium (DMEM) supplemented with 10% FBS at 37°C with 5% CO2 (thirty 150 mm plates per Cas12a). For each 150 mm plate transfection, 7 µg of pAAV9 serotype packaging plasmid, 20 µg of pDF6 helper plasmid, and 7 µg of AAV2 plasmid carrying the construct of interest were added to 1 mL of serum-free DMEM. To the mixture, we added 68 µL of Hieff Trans Liposomal Transfection Reagent and incubated it for 20 minutes at room temperature before adding it to the cells in each 150 mm plate68 µL of Hieff Trans Liposomal Transfection Reagent was added to the mixture and incubated for 20 minutes at room temperature, then added to each 150 mm plate. After 8 hours of incubation, 25 mL of warm maintenance medium was used to replace the old growth medium. Cells were harvested by scraping and pelleted by centrifugation 60 hours after transfection. The AAV2/9 viral particles were then purified from the pellet according to a previously described procedure[25]. We used the plasmid of each AAV-Cas12a-crRNA for the real-time PCR standards, the concentration of which was measured by absorbance at 260 nm. To calculate the copy number, we took into account the molecular weight of the plasmid and its measured concentration. We made serial dilutions of the standards in the range of 10^1^–10^-6^ ng per 1 µL using ddH2O. For the titration of AAV2/9 viral particles, we performed a real-time quantitative PCR-based method as described previously[26]. The standard curve showing the data points for the dilution series of the AAV-Cas12a-crRNA plasmid is presented (**Response Fig. 4B**). As shown in **Response Fig. 4C**, the titer of AAV-enEbCas12a-crRNA viral particles achieved 4.4×10^12^ total genome copies/mL.

*2) Which dose was actually delivered to the mice? It says 2e11 in the text but 5e11 in the figure, and I couldn't find additional information in the Methods.*

Response:

Thank you so much for your question. We are very sorry for our inadvertent error. AAV viral particles were administered intravenously to 5-6 week old male C57/BL6 mice via lateral tail vein injection at a dose of 2×10^11^ genomic copies per mouse. We have added additional information in the Methods section. "Changes Highlighted" can be found on lines 309-330 in the revised version. The text is as follows:

“AAV vial particles were administered intravenously to 5-6 week old male C57/BL6 mice via lateral tail vein injection at a dose of 2×10^11^ genomic copies per mouse. Prior to injection, the AAV dose was adjusted to 150 μL or 200 μL with sterile phosphate-buffered saline (PBS) (Gibco) at pH 7.4. All mice were maintained in the specific pathogen-free animal facility at Wuhan University and all animal experiments were approved by the Institutional Animal Care and Use Committee of Wuhan University. To track the serum levels of total cholesterol, we performed orbital blood sampling on mice at various time points. After allowing the blood to clot at room temperature, the serum was separated by centrifugation and stored at -20°C for subsequent analysis. Serum total cholesterol was measured using Infinity Cholesterol Reagent (Thermo Fisher) according to manufacturer's instructions. 1 × 1 × 3 mm^3^ liver was cut into pieces and then lysed for the indels analysis.”

*3) The drop in cholesterol levels in the mice is not overly impressive albeit significant. It would be good if the authors could further support and illustrate vector efficacy by showing the results of T7E1 assays from the mouse livers and quantifying the extent of target editing. Does it correlate with the mild drop in cholesterol?*

Response:

Thank you for your good advice. We have added the results of T7E1 assay from the mouse livers and quantifying the extent of target editing. The gel image of T7E1 is shown in **Response Fig. 4D**, and the average indel frequency is approximately 10%. We also performed the deep sequencing to validate the indels. As shown in **Response Fig. 4E**, enEbCas12a-*PCSK9* crRNA induced indels at the *PCSK9* genomic loci. *PCSK9* is a therapeutically relevant gene involved in cholesterol homeostasis[27]. Disruption of *PCSK9* is associated with reduced risk of cardiovascular disease and lower levels of low-density lipoprotein (LDL) cholesterol[15, 28-31]. In a similar way, by using AAV-enEbCas12a-*PCSK9* crRNA to disrupt the CDS region of *PCSK*, a reduction in cholesterol levels was observed. The destruction of *PCSK9* gene in the liver correlated with a slight decrease in cholesterol levels. This correlation is also similar to that found in previous reports[31]. This figure has been added in the revision of **S14 Fig.**

**Response** **Figure 4:** Evaluation of the All-in-One AAV-Cas12a-crRNA System. **A**, Single vector AAV system. **B**, Standard curve showing the data points for the dilution series of the AAV-Cas12a-crRNA plasmid. **C**, The titer of AAV-Cas12a-crRNA viral particles. **D**, *In vivo* genome editing using AAV-enEbCas12a-PCSK9-crRNA. Indels analyzed by T7E1 assay. (*n* = 3 animals for time points). **E**, Sequencing reads show representative mutations of AAV-enEbCas12a-PCSK9-crRNA mediated gene editing in liver. Dashes represent the DNA deletions. The number at the right side of each sequence is the length of indel (−, deletion).

Special thanks a lots for your good comments.

References

1.	Harrington LB, Burstein D, Chen JS, Paez-Espino D, Ma E, Witte IP, et al. Programmed DNA destruction by miniature CRISPR-Cas14 enzymes. Science (New York, NY). 2018;362(6416):839-42. Epub 2018/10/20. doi: 10.1126/science.aav4294. PubMed PMID: 30337455; PubMed Central PMCID: PMCPMC6659742.

2.	Karvelis T, Bigelyte G, Young JK, Hou Z, Zedaveinyte R, Budre K, et al. PAM recognition by miniature CRISPR-Cas12f nucleases triggers programmable double-stranded DNA target cleavage. Nucleic acids research. 2020;48(9):5016-23. Epub 2020/04/05. doi: 10.1093/nar/gkaa208. PubMed PMID: 32246713; PubMed Central PMCID: PMCPMC7229846.

3.	Wu Z, Zhang Y, Yu H, Pan D, Wang Y, Wang Y, et al. Programmed genome editing by a miniature CRISPR-Cas12f nuclease. Nature chemical biology. 2021;17(11):1132-8. Epub 2021/09/04. doi: 10.1038/s41589-021-00868-6. PubMed PMID: 34475565.

4.	Xu X, Chemparathy A, Zeng L, Kempton HR, Shang S, Nakamura M, et al. Engineered miniature CRISPR-Cas system for mammalian genome regulation and editing. Molecular cell. 2021;81(20):4333-45.e4. Epub 2021/09/05. doi: 10.1016/j.molcel.2021.08.008. PubMed PMID: 34480847.

5.	Kim DY, Lee JM, Moon SB, Chin HJ, Park S, Lim Y, et al. Efficient CRISPR editing with a hypercompact Cas12f1 and engineered guide RNAs delivered by adeno-associated virus. Nature biotechnology. 2022;40(1):94-102. Epub 2021/09/04. doi: 10.1038/s41587-021-01009-z. PubMed PMID: 34475560; PubMed Central PMCID: PMCPMC8763643 have filed patent applications on the gRNA-engineered Cas12f. Y.-S.K. and D.Y.K. are co-founders of GenKOre. The remaining authors declare that the research was conducted in the absence of any commercial or financial relationships that could be construed as a potential conflict of interest. The authors declare no competing nonfinancial interests.

6.	Kim DY, Chung Y, Lee Y, Jeong D, Park KH, Chin HJ, et al. Hypercompact adenine base editors based on transposase B guided by engineered RNA. Nature chemical biology. 2022;18(9):1005-13. Epub 2022/08/02. doi: 10.1038/s41589-022-01077-5. PubMed PMID: 35915259.

7.	Kong X, Zhang H, Li G, Wang Z, Kong X, Wang L, et al. Engineered CRISPR-OsCas12f1 and RhCas12f1 with robust activities and expanded target range for genome editing. Nature communications. 2023;14(1):2046. Epub 2023/04/12. doi: 10.1038/s41467-023-37829-7. PubMed PMID: 37041195; PubMed Central PMCID: PMCPMC10090079 H.Y. and Y.Z. are cofounders of HuiEdit Therapeutics Co., Ltd. (“HuiEdit”). Provisional patent applications PCT/CN2022/089053 and PCT/CN2022/142467 have been filed with inventors X.K., X.Q.K., and L.W. and owned by the patent applicant HuidaGene, claiming especially the CRISPR-Cas12 systems herein. The remaining authors declare no competing interests.

8.	Esvelt KM, Mali P, Braff JL, Moosburner M, Yaung SJ, Church GM. Orthogonal Cas9 proteins for RNA-guided gene regulation and editing. Nature methods. 2013;10(11):1116-21. Epub 2013/10/01. doi: 10.1038/nmeth.2681. PubMed PMID: 24076762; PubMed Central PMCID: PMCPMC3844869.

9.	Kleinstiver BP, Prew MS, Tsai SQ, Topkar VV, Nguyen NT, Zheng Z, et al. Engineered CRISPR-Cas9 nucleases with altered PAM specificities. Nature. 2015;523(7561):481-5. Epub 2015/06/23. doi: 10.1038/nature14592. PubMed PMID: 26098369; PubMed Central PMCID: PMCPMC4540238.

10.	Müller M, Lee CM, Gasiunas G, Davis TH, Cradick TJ, Siksnys V, et al. Streptococcus thermophilus CRISPR-Cas9 Systems Enable Specific Editing of the Human Genome. Molecular therapy : the journal of the American Society of Gene Therapy. 2016;24(3):636-44. Epub 2015/12/15. doi: 10.1038/mt.2015.218. PubMed PMID: 26658966; PubMed Central PMCID: PMCPMC4786917.

11.	Agudelo D, Carter S, Velimirovic M, Duringer A, Rivest JF, Levesque S, et al. Versatile and robust genome editing with Streptococcus thermophilus CRISPR1-Cas9. Genome research. 2020;30(1):107-17. Epub 2020/01/05. doi: 10.1101/gr.255414.119. PubMed PMID: 31900288; PubMed Central PMCID: PMCPMC6961573.

12.	Hou Z, Zhang Y, Propson NE, Howden SE, Chu LF, Sontheimer EJ, et al. Efficient genome engineering in human pluripotent stem cells using Cas9 from Neisseria meningitidis. Proceedings of the National Academy of Sciences of the United States of America. 2013;110(39):15644-9. Epub 2013/08/14. doi: 10.1073/pnas.1313587110. PubMed PMID: 23940360; PubMed Central PMCID: PMCPMC3785731.

13.	Lee CM, Cradick TJ, Bao G. The Neisseria meningitidis CRISPR-Cas9 System Enables Specific Genome Editing in Mammalian Cells. Molecular therapy : the journal of the American Society of Gene Therapy. 2016;24(3):645-54. Epub 2016/01/20. doi: 10.1038/mt.2016.8. PubMed PMID: 26782639; PubMed Central PMCID: PMCPMC4786937.

14.	Amrani N, Gao XD, Liu P, Edraki A, Mir A, Ibraheim R, et al. NmeCas9 is an intrinsically high-fidelity genome-editing platform. Genome biology. 2018;19(1):214. Epub 2018/12/07. doi: 10.1186/s13059-018-1591-1. PubMed PMID: 30518407; PubMed Central PMCID: PMCPMC6282386.

15.	Ran FA, Cong L, Yan WX, Scott DA, Gootenberg JS, Kriz AJ, et al. In vivo genome editing using Staphylococcus aureus Cas9. Nature. 2015;520(7546):186-91. Epub 2015/04/02. doi: 10.1038/nature14299. PubMed PMID: 25830891; PubMed Central PMCID: PMCPMC4393360.

16.	Friedland AE, Baral R, Singhal P, Loveluck K, Shen S, Sanchez M, et al. Characterization of Staphylococcus aureus Cas9: a smaller Cas9 for all-in-one adeno-associated virus delivery and paired nickase applications. Genome biology. 2015;16:257. Epub 2015/11/26. doi: 10.1186/s13059-015-0817-8. PubMed PMID: 26596280; PubMed Central PMCID: PMCPMC4657203.

17.	Najm FJ, Strand C, Donovan KF, Hegde M, Sanson KR, Vaimberg EW, et al. Orthologous CRISPR-Cas9 enzymes for combinatorial genetic screens. Nature biotechnology. 2018;36(2):179-89. Epub 2017/12/19. doi: 10.1038/nbt.4048. PubMed PMID: 29251726; PubMed Central PMCID: PMCPMC5800952.

18.	Tycko J, Barrera LA, Huston NC, Friedland AE, Wu X, Gootenberg JS, et al. Pairwise library screen systematically interrogates Staphylococcus aureus Cas9 specificity in human cells. Nature communications. 2018;9(1):2962. Epub 2018/07/29. doi: 10.1038/s41467-018-05391-2. PubMed PMID: 30054474; PubMed Central PMCID: PMCPMC6063963 consultants, or former employees of Editas Medicine. X.W. and O.O.A. declare no competing interests.

19.	Kim E, Koo T, Park SW, Kim D, Kim K, Cho HY, et al. In vivo genome editing with a small Cas9 orthologue derived from Campylobacter jejuni. Nature communications. 2017;8:14500. Epub 2017/02/22. doi: 10.1038/ncomms14500. PubMed PMID: 28220790; PubMed Central PMCID: PMCPMC5473640 and S.K. are employed by ToolGen, Inc. Je.H.K., S.W.P., D.W.S., E.K., and S.K. have filed patent applications based on this work. The remaining authors declare no competing financial interests.

20.	Yamada M, Watanabe Y, Gootenberg JS, Hirano H, Ran FA, Nakane T, et al. Crystal Structure of the Minimal Cas9 from Campylobacter jejuni Reveals the Molecular Diversity in the CRISPR-Cas9 Systems. Molecular cell. 2017;65(6):1109-21.e3. Epub 2017/03/18. doi: 10.1016/j.molcel.2017.02.007. PubMed PMID: 28306506.

21.	Edraki A, Mir A, Ibraheim R, Gainetdinov I, Yoon Y, Song CQ, et al. A Compact, High-Accuracy Cas9 with a Dinucleotide PAM for In Vivo Genome Editing. Molecular cell. 2019;73(4):714-26.e4. Epub 2018/12/26. doi: 10.1016/j.molcel.2018.12.003. PubMed PMID: 30581144; PubMed Central PMCID: PMCPMC6386616.

22.	Hu Z, Wang S, Zhang C, Gao N, Li M, Wang D, et al. A compact Cas9 ortholog from Staphylococcus Auricularis (SauriCas9) expands the DNA targeting scope. PLoS biology. 2020;18(3):e3000686. Epub 2020/04/01. doi: 10.1371/journal.pbio.3000686. PubMed PMID: 32226015; PubMed Central PMCID: PMCPMC7145270 following competing interests: The authors have applied for a patent related to this work.

23.	Schmidt MJ, Gupta A, Bednarski C, Gehrig-Giannini S, Richter F, Pitzler C, et al. Improved CRISPR genome editing using small highly active and specific engineered RNA-guided nucleases. Nature communications. 2021;12(1):4219. Epub 2021/07/11. doi: 10.1038/s41467-021-24454-5. PubMed PMID: 34244505; PubMed Central PMCID: PMCPMC8271026 K.K., A.K., A.S., A.N., S.S. have filed patent applications through Bayer AG, Casebia LLC, and CRISPR Therapeutics on the results described herein. A.C., W.M.C., M.G., K.K., A.G., M.L., S.J.S., C.P., M.S., J.T. hold stock or shares in the affiliated companies.

24.	Hu Z, Zhang C, Wang S, Gao S, Wei J, Li M, et al. Discovery and engineering of small SlugCas9 with broad targeting range and high specificity and activity. Nucleic acids research. 2021;49(7):4008-19. Epub 2021/03/16. doi: 10.1093/nar/gkab148. PubMed PMID: 33721016; PubMed Central PMCID: PMCPMC8053104.

25.	Zolotukhin S, Byrne BJ, Mason E, Zolotukhin I, Potter M, Chesnut K, et al. Recombinant adeno-associated virus purification using novel methods improves infectious titer and yield. Gene therapy. 1999;6(6):973-85. Epub 1999/08/24. doi: 10.1038/sj.gt.3300938. PubMed PMID: 10455399.

26.	Veldwijk MR, Topaly J, Laufs S, Hengge UR, Wenz F, Zeller WJ, et al. Development and optimization of a real-time quantitative PCR-based method for the titration of AAV-2 vector stocks. Molecular therapy : the journal of the American Society of Gene Therapy. 2002;6(2):272-8. Epub 2002/09/28. doi: 10.1006/mthe.2002.0659. PubMed PMID: 12349826.

27.	Fitzgerald K, Frank-Kamenetsky M, Shulga-Morskaya S, Liebow A, Bettencourt BR, Sutherland JE, et al. Effect of an RNA interference drug on the synthesis of proprotein convertase subtilisin/kexin type 9 (PCSK9) and the concentration of serum LDL cholesterol in healthy volunteers: a randomised, single-blind, placebo-controlled, phase 1 trial. Lancet (London, England). 2014;383(9911):60-8. Epub 2013/10/08. doi: 10.1016/s0140-6736(13)61914-5. PubMed PMID: 24094767; PubMed Central PMCID: PMCPMC4387547.

28.	Abifadel M, Varret M, Rabès JP, Allard D, Ouguerram K, Devillers M, et al. Mutations in PCSK9 cause autosomal dominant hypercholesterolemia. Nature genetics. 2003;34(2):154-6. Epub 2003/05/06. doi: 10.1038/ng1161. PubMed PMID: 12730697.

29.	Cohen J, Pertsemlidis A, Kotowski IK, Graham R, Garcia CK, Hobbs HH. Low LDL cholesterol in individuals of African descent resulting from frequent nonsense mutations in PCSK9. Nature genetics. 2005;37(2):161-5. Epub 2005/01/18. doi: 10.1038/ng1509. PubMed PMID: 15654334.

30.	Horton JD, Cohen JC, Hobbs HH. Molecular biology of PCSK9: its role in LDL metabolism. Trends in biochemical sciences. 2007;32(2):71-7. Epub 2007/01/12. doi: 10.1016/j.tibs.2006.12.008. PubMed PMID: 17215125; PubMed Central PMCID: PMCPMC2711871.

31.	Liu Z, Chen S, Xie W, Song Y, Li J, Lai L, et al. Versatile and efficient in vivo genome editing with compact Streptococcus pasteurianus Cas9. Molecular therapy : the journal of the American Society of Gene Therapy. 2022;30(1):256-67. Epub 2021/06/27. doi: 10.1016/j.ymthe.2021.06.013. PubMed PMID: 34174445; PubMed Central PMCID: PMCPMC8753289.
